# Adaptive Multi-Channel Clustering in IEEE 802.11s Wireless Mesh Networks †

**DOI:** 10.3390/s21217215

**Published:** 2021-10-29

**Authors:** Michael Rethfeldt, Tim Brockmann, Benjamin Beichler, Christian Haubelt, Dirk Timmermann

**Affiliations:** Institute of Applied Microelectronics and Computer Engineering, Faculty of Computer Science and Electrical Engineering, University of Rostock, 18051 Rostock, Germany; tim.brockmann@uni-rostock.de (T.B.); benjamin.beichler@uni-rostock.de (B.B.); christian.haubelt@uni-rostock.de (C.H.); dirk.timmermann@uni-rostock.de (D.T.)

**Keywords:** WLAN, wireless mesh network, IEEE 802.11s, hybrid wireless mesh protocol, Airtime Link Metric, clustering, channel assignment

## Abstract

WLAN mesh networks are one of the key technologies for upcoming smart city applications and are characterized by a flexible and low-cost deployment. The standard amendment IEEE 802.11s introduces low-level mesh interoperability at the WLAN MAC layer. However, scalability limitations imposed by management traffic overhead, routing delays, medium contention, and interference are common issues in wireless mesh networks and also apply to IEEE 802.11s networks. Possible solutions proposed in the literature recommend a divide-and-conquer scheme that partitions the network into clusters and forms smaller collision and broadcast domains by assigning orthogonal channels. We present CHaChA (Clustering Heuristic and Channel Assignment), a distributed cross-layer approach for cluster formation and channel assignment that directly integrates the default IEEE 802.11s mesh protocol information and operating modes, retaining unrestricted compliance to the WLAN standard. Our concept proposes further mechanisms for dynamic cluster adaptation, including subsequent cluster joining, isolation and fault detection, and node roaming for cluster balancing. The practical performance of CHaChA is demonstrated in a real-world 802.11s testbed. We first investigate clustering reproducibility, duration, and communication overhead in static network scenarios of different sizes. We then validate our concepts for dynamic cluster adaptation, considering topology changes that are likely to occur during long-term network operation and maintenance.

## 1. Introduction

The wireless local area network (WLAN) standard amendment IEEE 802.11s [1,2] integrates basic mesh functions directly into the WLAN link layer, providing the basis for interoperable mesh solutions to realize smart city applications [3,4,5]. For this purpose, 802.11s defines mandatory mechanisms for spontaneous networking and message forwarding (routing) between mesh nodes. Above the standardized basic functions, however, numerous requirements and problems exist in order to be able to implement the operation of 802.11s mesh networks efficiently and reliably, especially with increasing complexity.

Scalability limitations of WLAN mesh networks (WMN) especially arise from their inherently limited effective channel utilization [6,7,8]. Devices within radio range on the same channel compete for exclusive access to the medium and thus form a collision domain. The totality of all nodes connected on the same channel is separately referred to as a broadcast domain, since it is traversed in its entirety when broadcast messages are transmitted. However, unicast message forwarding across multiple hops also requires the corresponding number of individual transmissions, so that a mesh path traverses multiple collision domains. This is accompanied by the overhead traffic of the WLAN link layer, which is generated, e.g., by periodic announcements (beacons) of the mesh nodes or in the context of the routing protocol. Consequently, the communication latency and transmission error probability inevitably increase with the number of nodes and average path length in the network.

These constraints significantly determine the performance of services and applications in the mesh network. They must therefore be considered and dealt with as part of the network orchestration and management. An established methodology for scalable network structuring and the approach of many research works is to partition the mesh network into independent regions, called clusters. This is done, for example, by choosing appropriate cluster heads, each of which is responsible only for managing its individual cluster [9,10,11]. In combination with clustering approaches, channel selection strategies can result in more efficient use of the available spectrum [12,13,14]. The WLAN technology mainly uses channels in unlicensed ISM bands in the 2.4 and 5 GHz frequency range. Neighboring clusters configured on non-overlapping (orthogonal) channels always operate in separate collision domains, regardless of their physical proximity. For this purpose, the clusters must have sufficient frequency spacing, which depends on the selected channel bandwidth. As a result, media access can take place without mutual interference and thus completely in parallel. A combined approach to cluster formation and channel selection promises more efficient use of communication resources by distributed applications, especially if they can be appropriately mapped to the resulting clusters. In a previous work by the authors, a centralized monitoring solution for 802.11s networks was developed, where a dedicated node collects status information of all other nodes [15]. However, as network size and mesh path length increase, the application becomes increasingly inefficient. Moreover, the central node represents a bottleneck and Single Point of Failure. Practical research has shown that a decentralized approach is necessary to ensure the scalability and resilience of the monitoring solution [16]. Thus, the task of status monitoring could be implemented in a decentralized manner by multiple cluster heads.

**Figure 1:**
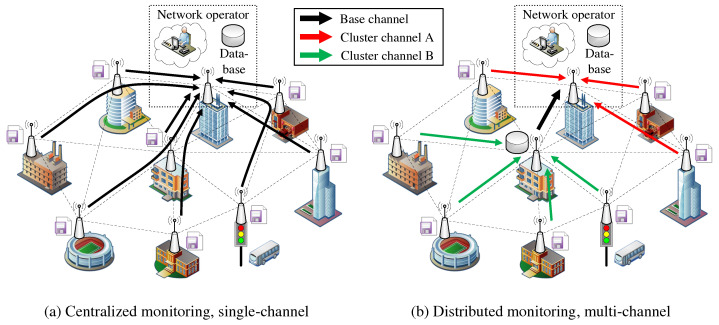
Distributed status monitoring in a smart city scenario. Reprinted from ref. [18].

The example in Figure 1 contrasts a centralized data retrieval in Figure 1a with a decentralized approach in Figure 1b that uses clusters on orthogonal channels. Here, the centrally positioned node acts as a second cluster head, fetching information for its part of the network (green transmissions) in parallel with the data query of the administrative access node (red transmissions). Assuming at least two WLAN interfaces per mesh node, which allow parallel communication on separate channels within the backbone, the data collected in the cluster can be synchronized on an independent base channel. Compared to the centralized single-channel case, the path lengths of the transmissions are shortened while communication parallelism and resilience are increased.

Consequently, this paper presents a distributed clustering approach that preserves full compatibility with the standard 802.11s specification without introducing any modifications to the WLAN protocol stack. The developed solution CHaChA (Clustering Heuristic and Channel Assignment) integrates the link and path information of the 802.11s link layer to derive topology information and use it directly for cluster formation and channel selection. CHaChA increases the scalability of common single-channel 802.11s networks by efficiently employing additional mesh interfaces to create independent sub-networks (clusters) on orthogonal channels. Thereby, unrestrained network-wide connectivity is ensured on a base channel via a dedicated interface. The created clusters, featuring smaller collision and broadcast domains, allow for the isolation of distributed applications, thereby increasing overall communication parallelism and relieving stress on the base channel.

This article considerably extends a previous work [17] where we introduced the basic concepts of CHaChA and executed an early prototype evaluation. Using the status monitoring of mesh networks as application example, the general performance advantages resulting from clustering were demonstrated. The work at hand is largely based on further results of the corresponding author’s PhD thesis [18], not yet published in any conference or journal. It first presents the detailed concepts for initial clustering and channel selection in IEEE 802.11s mesh networks. We substantially extend CHaChA by mechanisms for cluster adaptation to dynamic topology changes that are likely to occur during long-term network operation and maintenance. These mechanisms include automatic cluster joining of subsequently installed nodes, isolation and fault detection, and node roaming for cluster balancing. The concepts are implemented as software prototype and are practically validated in a 25-node real-world mesh testbed.

The remainder of this article is organized as follows: Section 2 outlines the basic principles of the IEEE 802.11s WLAN mesh specification and its Linux implementation. In Section 3, we classify and discuss related work in the area of combined clustering and channel selection for WLAN mesh networks. Section 4 illustrates the extended concepts of our cross-layer approach CHaChA. In Section 5, we describe our practical prototype implementation, which is thoroughly evaluated in Section 6. Finally, we give a summary and conclusion in Section 7 and briefly state future research directions.

## 2. IEEE 802.11s WLAN Mesh Networks

As the first common industry WLAN mesh standard, the amendment IEEE 802.11s was ratified in September 2011 [1]. It enables vendor-independent infrastructure-less multi-hop communication based on the widespread 802.11 technology. Mesh functions such as peering and routing are directly integrated into the MAC layer specification. Thus, 802.11s comes as a promising alternative to former, non-interoperable network-layer mesh routing protocols. To ensure interoperability, every 802.11s node must support the Hybrid Wireless Mesh Protocol (HWMP) and Airtime Link Metric (ALM) for mesh routing [19]. The default reactive mode of HWMP is based on the Ad Hoc On-Demand Distance Vector (AODV) routing protocol and determines a path as soon as it is needed. As usual for distance vector protocols, a node using HWMP only maintains links to nodes within radio range (neighbors) and paths to selected destinations in multi-hop distance, to which communication has been explicitly initiated, e.g., by higher layers. A node’s path list maintains the forwarding rules to destination nodes. To every destination, only the past via the best neighbor (“next hop”) is kept, resulting in the smallest ALM cost metric. Path information is updated periodically unless it expires with an inactivity timeout.

Optionally, HWMP supports using a tree-based proactive routing mode alongside the reactive mode. Nodes that will be contacted frequently can use this mode to periodically announce themselves as so-called root nodes in the network and enforce determination of uni- or bidirectional path information to them in advance. This reduces routing latency at the expense of additional message overhead [19]. There are three proactive routing variants: In the mode “proactive PREQ without PREP”, a root node periodically sends PREQ (path request) broadcast messages addressed to all nodes in the network. However, no response via a PREP (path reply) is expected, so all nodes simply create the reverse path to the root node. If this is sufficient for the use case, this mode generates the lowest overhead. The mode “proactive PREQ with PREP” is similar to the previous variant, but a PREP is required from each node. This means that bidirectional paths are always maintained between the root node and all other nodes. The mode “proactive RANN” represents a compromise between the first two variants. Here, so-called RANN (route announcement) messages are sent periodically, but these only propagate or update the ALM cost metric to the root node in the network. Bidirectional path creation using unicast PREQ and PREP is initiated by the receiving mesh nodes only when path information changes.

The ALM estimates the time cost for frame transmissions considering protocol overhead, data rate, and error probability. Each node only calculates the ALM to its neighbors, and individual link costs are accumulated and disseminated during path discovery. Due to this cumulative characteristic, the ALM of a multi-hop path represents the overall average estimated time to transmit a single frame from source to destination. The airtime cost $c_{a}$ (in μs) is calculated per link, as defined by the IEEE 802.11 standard [1]:

(1) $$c_{a}=\;\left[O+\frac{B_{t}}{r}\right]\;\cdot \;\frac{1}{1-e_{fr}}$$

*O* is a constant for the channel access and MAC protocol overhead. $B_{t}$ is the test frame size, specified as 1 kB in the standard. *r* denotes the test frame data rate, given in Mbps, whereas $e_{fr}$ denotes the expected frame error rate. The estimation of $e_{fr}$ and the value *O* are not predefined by the 802.11s standard but left open to vendor implementations.

The Linux kernel module mac80211 [20] currently contains the most sophisticated implementation of 802.11s and HWMP. By default, path information expires after 5 s and are refreshed 1 s in advance of a timeout. In proactive mode, a node announces itself every 5 s via broadcast, triggering a path discovery or update on all other nodes. Since some parameters in ALM calculation are left open to vendor implementations, Linux provides own variants for error rate estimation and overhead constants. While *O* is set to the constant value 1, data rate *r* is estimated by the rate control algorithm. The error rate $e_{fr}$ is updated on every frame transmission and calculated by a moving average filter. Depending on the WLAN hardware and driver, this estimation is often provided by the rate control algorithm as well.

## 3. State of the Art and Related Works

In the area of WMN management, various research works exist that propose a decentralized organization of the network by partitioning it into logical subnetworks (clusters), but do not address the aspect of channel selection for the resulting groups of nodes. The motivation is primarily to distribute management tasks to increase their scalability. With ANMP (Ad Hoc Network Management Protocol) [21] and Guerrilla [22], graph-theoretical methods for hierarchical clustering were described with the goal of reducing the traffic generated by status monitoring of the nodes based on the Simple Network Management Protocol (SNMP). Another solution called ABCP (Access-Based Clustering Protocol) [23] requires strong adaptations at the WLAN link layer. Mesh-Mon [24] represents a broadcast-based clustering protocol that is also used to distribute an SNMP monitoring solution. MeshMan [25] defines another protocol based on ICMP that performs hierarchical addressing and clustering of nodes. None of the aforementioned work takes the WLAN mesh specification 802.11s into account. ANMP, Guerrilla, and ABCP have also only been studied simulatively.

**Figure 2:**
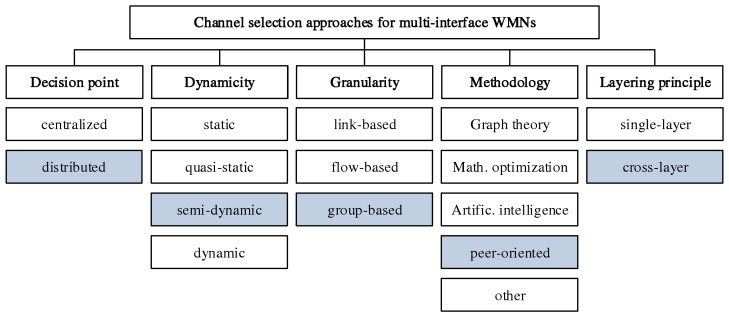
Classification of channel selection approaches acc. to Islam et al. [12] (attributes of own work CHaChA highlighted in blue). Reprinted from ref. [18].

In the research field of channel selection for WLAN mesh environments, greater methodological commonalities exist in the form of group-based approaches. The surveys [12,13,14] provide an overview of the large number of contributions in this area. While no approaches considering 802.11s are discussed, the survey [12] presents a helpful classification of channel selection strategies for WMN with multiple network interfaces per device. This is shown in Figure 2, and the classification of our own work CHaChA is highlighted. According to [12], the following criteria are distinguished:

**Decision point:** A basic feature is the decision point for channel selection. A distinction is made between centralized and distributed approaches, depending on whether the responsibility lies with a dedicated entity (usually with global network knowledge) or with many or all nodes. Distributed approaches usually have higher scalability and resilience. In addition, they are independent of global knowledge and can be implemented using only local information, such as connections to neighboring nodes or forwarding rules to selected endpoints [13].
**Dynamicity:** A second criterion is dynamicity, i.e., the ability to respond to changes in the network. This ranges from purely static and quasi-static methods, which perform a configuration only once when the network is put into operation or regularly at large intervals, to semi-dynamic methods, which can make new decisions within shorter periods or in response to specific events, to fully dynamic strategies, which also allow dealing with mobile nodes.
**Granularity:** The granularity of the method defines whether different channels are chosen for individual connections (links), end-to-end transmissions (flows) or groups of nodes. The latter category also includes clustering approaches, which represent a trade-off between the performance gain achievable through fine-granular channel separation and the message overhead caused by frequent reconfiguration.
**Methodology:** Existing methods particularly differ in the way they solve the channel selection problem. Centralized approaches are often based on graph theory (e.g., graph coloring), mathematical optimization techniques (e.g., linear programming), or artificial intelligence (e.g., evolutionary algorithms). They usually require global knowledge about the network and often involve high computational overhead. On the other hand, many decentralized approaches work peer-oriented and mainly use local connection information of the nodes.
**Layering principle:** Another feature for distinguishing channel selection strategies is whether they are located only at a single layer of the network protocol stack (single-layer approaches) or operate across multiple layers (cross-layer approaches). The latter often offer higher optimization potential by using information from different layers. However, depending on the availability of practical interfaces for information exchange, there may be greater challenges with regard to implementing the methods and integrating them into existing systems.

The focus of our own work CHaChA lies on a group-based channel selection in the form of a clustering approach, which simultaneously pursues the goal of application distribution and more efficient utilization of the frequency spectrum. The decentralized, semi-dynamic, and peer-oriented approach is implemented at the application layer and integrates local connection information of the 802.11s link layer according to the cross-layer principle without making any changes to the network protocol stack. Another important objective was to implement a prototype and prove its practicality.

Table 1 contrasts CHaChA with related research papers that also present combined approaches to clustering and channel selection. Older solutions without integration of 802.11s, already discussed in the surveys [12,13,14], are listed as well as more recent contributions that consider 802.11s at least in a preliminary or modified form. The approaches are grouped according to the constellation of the following requirements: the consideration of 802.11s, the decentralization of the approach, and its investigation in a real test environment.

Most of the earlier work done before 802.11s was specified and therefore does not use its routing mechanisms and connection information. All of the work was also studied only simulatively. Nevertheless, the contributions CCA [30] and ISC [27], whose concepts regarding a dedicated base channel (common channel) as well as the metric “Highest Connectivity” were also integrated into CHaChA, deserve special mention.

Only the works [9,10,11] consider 802.11s in their studies. The graph-theoretic approach CGCA [9] uses centralized clustering depending on a mesh gateway that obtains global network knowledge by communicating with all nodes. CGCA also requires position information of the nodes and excludes topology changes. The distributed approach of Kapse et al. [10] declares mesh gateways as cluster heads regardless of their topological fitness. Cluster membership of nodes is determined using hop distance. However, the 802.11s routing mechanism Hybrid Wireless Mesh Protocol (HWMP) uses the Airtime Link Metric (ALM) by default, which takes into account not only the length of a path, but also its average data rate and error probability. The work JRCAP [11] presents a combination of decentralized clustering-based channel selection and a routing protocol. The HWMP and ALM are replaced, so there is no interoperability with default 802.11s. A mesh gateway is required to implement the custom routing protocol. In addition, since no basic connectivity is ensured over a common channel, relay nodes between clusters are required. However, partial concepts of JRCAP for cluster roaming are considered in the context of CHaChA. The approaches [9,10,11] were only investigated simulatively above preliminary 802.11s models and do not integrate HWMP information.

In contrast to the related works, CHaChA represents a distributed solution that simultaneously preserves interoperability with standard 802.11s and integrates its local connection information directly via existing operating system interfaces. In addition, CHaChA has been studied as a prototype in a real test environment.

## 4. The CHaChA Clustering Approach

### 4.1. Overview and Terminology

The presented approach CHaChA includes, on the one hand, concepts for initial cluster formation and channel assignment when commissioning an 802.11s network. On the other hand, mechanisms for automatic cluster adaptation in case of subsequent changes to the network structure are presented. Our concept assumes nodes with multiple physical WLAN interfaces, at least two of which are available for use within the mesh network. This represents a common hardware configuration for modern WLAN devices, as can be expected especially in more complex backbone installations [12]. Of the interfaces, one remains as a dedicated primary interface on a given base channel for inter-cluster communication, while a secondary interface is reserved for intra-cluster communication. This principle, also known as “common channel”, is used in many research works to ensure unrestricted connectivity in the network at all times regardless of the cluster configuration [11,27]. Since all nodes are always reachable via the base channel, the primary interface is used to exchange any CHaChA control messages. In addition, its 802.11s link and path tables serve as basis for deriving topology information and metrics for cluster formation. Our simplifying assumption is that the physical parameters of the secondary interfaces are similar to those of the primary interface and that clustering decisions made based on its metrics are applicable in general.

**Figure 3:**
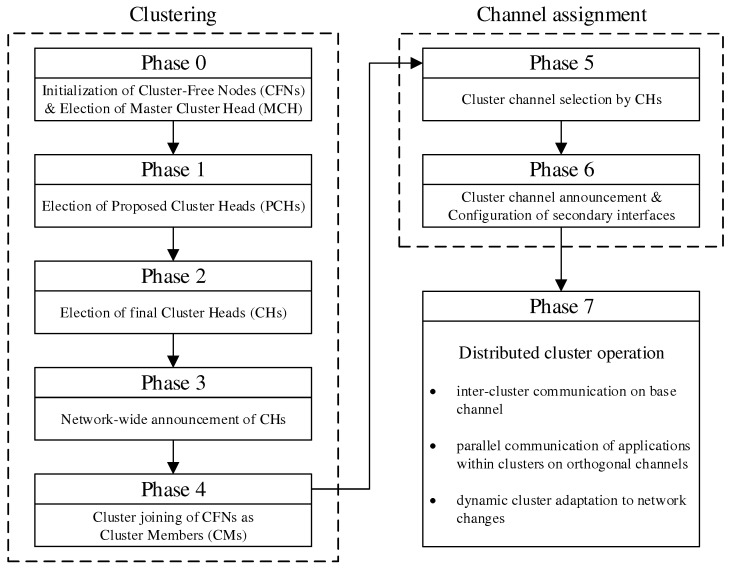
Phase sequence for initial clustering and channel assignment. Reprinted from ref. [18].

The initial cluster formation and channel assignment during mesh network commissioning are realized in a sequence of phases. Along the way, the mesh nodes take on different roles, which are summarized in Table 2. Figure 3 shows the overview of all steps. A detailed algorithmic description is given in Section 4.3.

Initial cluster formation occurs in phases 0–4, which divide the network into non-overlapping regions, taking into account the mesh topology. From all nodes starting as Cluster-Free Nodes (CFNs) at the beginning, temporary Proposed Cluster Heads (PCHs) emerge during the phases and compete as candidates for the final role as Cluster Head (CH). Each cluster consists of a lead CH and multiple Cluster Members (CMs) and forms a separate mesh network on the secondary interface of its participants. Compared to the base network on the primary interface, the individual clusters represent broadcast domains of smaller size. This has the potential to reduce the communication load on the base network by moving applications to appropriate clusters.

Phases 5 and 6 realize the cluster channel selection and configuration of the nodes’ secondary interfaces. The separation into orthogonal channels avoids interference between neighboring clusters as well as with the base channel, thus favoring parallelism of transmissions throughout the network. The current concept is limited to the integration of two WLAN interfaces per node. However, the strategy described can be extended analogously to scenarios in which additional interfaces are available for mesh communication. For example, applications within a cluster could be specifically assigned to different interfaces on orthogonal channels.

In phase 7, initialization of the network is complete, and distributed applications can benefit from the created clusters. Further mechanisms allow the resulting initial cluster constellation to respond to connectivity problems and moderate topology changes, such as subsequent installation, relocation, or removal of individual nodes in the course of maintenance activities. In this case, required incremental cluster adjustments are automatically handled by the CHs and CMs, respectively, while new nodes can find and join the existing clusters. However, in the case of detection of severe changes in the network structure and number of participants, it may be necessary to initiate a new cluster formation. The associated concepts beyond the initial phase sequence are discussed in Section 4.4.

The coordination of the phase sequence is taken over by a Master Cluster Head (MCH) elected by all nodes in phase 0. Preferably, the MCH has a central position in the network and thus a small average distance to the other nodes and initiates the phase transitions via control messages. After reaching phase 7, this coordinating task of the MCH expires, and it remains in the network as conventional CH. The design decision for a phase sequence controlled by messages offers the advantage of loosely synchronizing the timing of the mesh nodes without requiring a precise common time base. This would be necessary, e.g., for frequency hopping approaches, in which nodes must meet at fixed time intervals on rendezvous channels to negotiate channel sequences [12,13,14]. In particular, phase-based synchronization allows stabilization of the topology information and metrics obtained during each phase duration, on the basis of which cluster formation and channel selection decisions are made. In contrast to completely asynchronous methods, the phase-based approach can also be easily reproduced and analyzed in practice.

### 4.2. Metrics, Messages, and Parameters

A main motivation of CHaChA is its vendor-independent applicability above standard hardware and software. It is based on the unmodified Linux 802.11(s) protocol stack, which ensures mesh interoperability already at the link layer. Due to the 802.11s routing protocol HWMP, being a distance vector protocol, each participant has only incomplete knowledge about the network topology. This work therefore takes a heuristic approach, relying on the nodes’ local 802.11s connection information and deriving further metrics from it. Several metrics are used in CHaChA, which are first explained independently of the phase flow. Table 3 gives an overview of all metrics and their calculation.

**Airtime Link Metric (ALM):** The ALM is the default metric of the 802.11s routing protocol HWMP. It describes the time cost of a frame transmission to a destination node, taking into account the WLAN transmission rate and error probability. Due to its cumulative computation along all hops of a path, it represents both its length and overall quality. In CHaChA, the ALM serves as a distance measure, e.g., for determining nearby CHs.
**Centrality (CENT):** The CENT metric is an estimate of the centrality of a node’s position in the network. The node with largest CENT value ($CENT_{max}$) is initially assigned the MCH role. In mesh networks consisting of devices with similar WLAN hardware characteristics (number of antennas and supported transmission rates), the ALM cost of a path arises significantly from its hop count, provided that individual link conditions (e.g., strongly varying distance or attenuation characteristics) do not translate into a permanently high error rate and low data rate of individual hops. Consequently, the assumption can be made that the node positioned at the center of any mesh topology likely has the lowest ALM average of all paths to other nodes, since it reaches them via the shortest average path lengths. After a node has determined path information to all other nodes, for example by temporarily activating the proactive mode of the 802.11s routing protocol HWMP, it can calculate its centrality using the formula $CENT={\left(\sum_{i=1}^{\#paths}ALM_{i}\right)}^{-1}$.
**Network Size***N***:** As a result of a path establishment in proactive HWMP mode, a node simultaneously obtains knowledge about the overall number of participants in the network, denoted by *N*. It is obtained from the sum N=1+#(paths).
**Neighbor Count (NC):** The NC of a mesh node is given by the number of active links to neighboring nodes. Nodes with maximum NC in their neighborhood can reach the highest proportion of other nodes directly and are least likely to be isolated in their network region. For these reasons, they are considered suitable candidates for the CH role and compete for it as temporary PCHs. The NC metric has also been proposed under similar names in other research, such as Highest Connectivity (HC) in [27,30].
**PCH Neighbor Count (PCHNC):** The PCHNC is part of the calculation of the NC-to-PCHNC Ratio (NPR) and is only determined by PCH nodes. It denotes the number of mesh neighbors that are currently also in the PCH role due to the same degree of connectivity (NC metric) and competition for becoming final CH.
**NC-to-PCHNC Ratio (NPR):** The NPR is part of the metric Weighted NC-to-PCHNC Ratio (WNPR) and is calculated as the ratio of NC and PCHNC. All PCHs calculate it according to the rule $NPR=\frac{NC}{(1+PCHNC)\cdot N}$. To guarantee a range of values between 0 and 1, the metric is also normalized to the network size *N*. In the course of their competition, PCHs with higher NPR should be given priority since they are located in regions with fewer CH candidates. However, if NPR alone were used as a criterion, PCHs in the immediate vicinity of the outermost edge nodes of the network would be particularly preferred. Since these edge nodes usually have a lower connectivity degree (NC metric) than their neighbors located further within the network, they are not PCH candidates themselves. To compensate for an uncontrolled shift of final CHs towards the network edge, a centrality weight is added to the NPR in the form of the WNPR.
**Weighted NC-to-PCHNC Ratio (WNPR):** All PCHs compete for the CH role with this metric and compute it according to the rule $WNPR=NPR\;\cdot \frac{CENT}{CENT_{max}}$. Hence, it is obtained as the product of NPR and centrality of a PCH. For WNPR comparability between different PCHs, their own centrality is normalized to that of the MCH node ($CENT_{max}$), which was determined in phase 0. By means of the centrality weight, the competition finds a trade-off between favoring PCHs in underpopulated regions and keeping proximity to the MCH at the center of the network. On the one hand, this compensates for the dominance of PCHs close to the network edge, which is inherently sparse with PCH candidates as a result of the NPR metric. On the other hand, distributed applications can benefit from a proximity of CHs to the MCH, e.g., when the MCH acts as administrative synchronization point and central data sink on the base channel.
**Integer value of MAC address:** In case of a tie (two compared metric values are identical), the node with larger integer value of its MAC address wins. This allows for quick and clear decision-making in the different competitive phases of CHaChA. An exception is the NC metric, where a tie is explicitly allowed. As a result, several CH candidates are initially created, which are subsequently thinned out using the WNPR.

All CHaChA control messages are listed in Table 4. These include unicast messages, which are primarily exchanged between neighboring nodes, and broadcast messages for disseminating information throughout the network. Unlike unicast frames, the WLAN link layer transmits broadcast frames unacknowledged and thus without retransmissions in case of failure. To increase reliability, the CHaChA concept is to always send broadcast messages multiple times at the application layer. This concerns the CENT messages, carrying the CENT metric, as well as the PHASE_X messages of the MCH for announcing phase transitions. The cluster announcement messages of the CH nodes are already generated periodically. Various time interval and threshold parameters also allow for customization in terms of reliability and timing. Table 5 gives an overview of the CHaChA parameters.

### 4.3. Initial Clustering and Channel Assignment

The initial cluster formation is described in detail below, attributing all messages and parameters to the phase sequence. An exception are the message types NH2CH, JOIN_REQ/RESP, and LEAVE_REQ/RESP. These are used only in phase 7 for further concepts, which are explained in Section 4.4.

**Figure 4:**
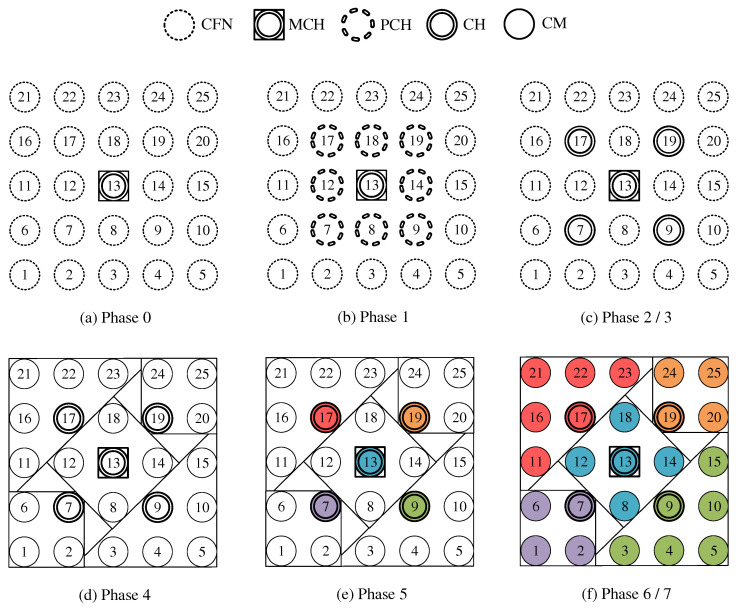
Intermediate phase results in an example network (mesh links between grid neighbors not shown for simplification; cluster channels of secondary interfaces highlighted by different colors). Reprinted from ref. [18].

**Phase 0—Network Initialization and MCH Election:** In phase 0, all nodes start as Cluster-Free Node (CFN) and are connected by their primary interface. A precondition for the execution of the CHaChA algorithm is the presence of at least one active connection to a neighboring node on the base channel. In the following, a synchronous start of execution is assumed on all devices, so that they run through the initial phase sequence together. Mechanisms for subsequently joining an existing cluster constellation are also located in phase 0 and discussed in Section 4.4.1.
  During phase 0, each CFN periodically sends NC unicast messages to its neighboring nodes at intervals of NC_PERIOD. These contain the current NC metric, which all nodes later compare in phase 1 to determine the PCHs. Until reaching phase 3 each CFN also activates the proactive routing mode of the HWMP to temporarily determine mesh paths to all other nodes. CHaChA makes targeted use of the proactive HWMP variants. At this stage, the most efficient of the three proactive modes is chosen (“proactive PREQ without PREP”), whereby a node in the network announces itself but does not expect a response for bidirectional path establishment. This variant is sufficient since all CFNs activate the mode and thus receive unidirectional path costs to each other. Compared to the conventional reactive HWMP mode, this temporarily generates more 802.11s control messages, but results in lower overhead than higher-layer network exploration (e.g., using ping scan) [34,35].
  As a result, each CFN knows the current network size *N* as well as the ALM cost to all other nodes. This enables computation of the CENT metric, and all nodes then announce to the network periodically at CENT_PERIOD intervals using CENT broadcast messages, thereby competing for the MCH role. Upon receiving messages with larger metric (or larger MAC address in case of a tie), nodes decide to withdraw from the competition and stop sending messages. Once the CFN with largest CENT value has sent at least CENT_THRESH consecutive messages without receiving any in return, it automatically becomes MCH. It later acts as CH of a central cluster and coordinates the remaining phase sequence. Its central position in the network and therefore lowest average distance to all other nodes results in minimal frame forwarding overhead when sending control messages and enables fast broadcast delivery. Figure 4a shows the network state at the end of phase 0 in the example of a 5 × 5-node grid topology. To announce the transition to phase 1 to all nodes, the MCH (node 13 in the example) broadcasts PHASE_1 messages at the time interval of PHASE_1_PERIOD. After a number of PHASE_1_TRIES broadcasts, it moves to the next phase itself. This procedure applies analogously to phases 1–6.
**Phase 1—PCH Election:** In phase 1, the election of CH candidates, called PCHs, takes place. For this purpose, all nodes compare their NC metric with the NC values of their neighbors exchanged in phase 0. The nodes with highest NC in their neighborhood switch from the CFN to the PCH role, while a tie in metrics is allowed. This can temporarily create multiple neighboring CH candidates with the same degree of connectivity, which are further thinned out in the subsequent phase. All PCHs now inform their neighboring nodes of their candidacy by sending PCH unicast messages. Figure 4b shows the intermediate result at the end of phase 1 in the example network, in which eight PCHs emerge. After the expiration of a waiting time defined as PHASE_2_DELAY (analogous parameters for phases 3–5), the MCH initiates the phase transition with PHASE_2 messages.
**Phase 2—CH Election:** All PCHs compute their WNPR metric at the beginning of this phase and send it via WNPR unicast messages to PCHs in their neighborhood. The PCHs with highest metric, or largest MAC address in case of metric tie, win the competition and become the final CHs. The losing PCHs switch back to the initial CFN role. Figure 4c shows the intermediate result at the end of phase 2 in the example network, with four CHs emerging in addition to the central MCH. After the PHASE_3_DELAY waiting time expires, the MCH initiates the transition to phase 3.
**Phase 3—CH Announcement:** All CFNs now switch from proactive routing to the more resource-saving reactive HWMP mode. Only the MCH and CHs remain in proactive mode to ensure that all other nodes have path information and ALM costs to them at all times. Furthermore, the MCH and CHs enable periodic broadcast of CH messages to announce their role and cluster information on the base channel. This includes the unique mesh ID as well as the WLAN channel of the cluster, for which joining nodes need to configure their secondary interfaces. Since 802.11s already implements mesh routing at the link layer, multi-hop communication of applications is also possible with link-local IPv6 addresses, which can be derived directly from the MAC addresses of the network interfaces [36]. For this reason, we do not discuss further concepts for the IP configuration of the nodes’ primary and secondary interfaces. In phase 3, i.e., before cluster joining of the CFNs and subsequent channel selection, the CH announcement messages carry only the mesh ID of the cluster, which corresponds to the MAC address of the respective CH. After the PHASE_4_DELAY waiting period, the MCH initiates the transition to phase 4.
**Phase 4—CFN Cluster Joining:** All CFNs determine the closest CH in terms of ALM path cost, join its cluster, and move to the node role CM. Figure 4d shows the intermediate result at the end of phase 4 in the example network. After expiry of the PHASE_5_DELAY waiting time, the MCH initiates the transition to phase 5. CFNs prioritize CHs in their neighborhood when choosing their cluster. Moreover, if the MCH is among them, it is chosen preferentially over other neighboring CHs. This strategy implies advantages for administrative applications, such as decentralized status monitoring of all nodes. Here, the MCH can serve as a synchronization point in a central position, to which a regular transmission of information from the other CHs takes place, thus providing a global network view. This eliminates the need to exchange information from those nodes that already belong to the cluster of the MCH.
  To join a cluster, a CFN sends a JOIN unicast message to the corresponding CH, which then adds it to its CM participant list. Analogous to the JOIN message, CHaChA defines a LEAVE message and associated handshake messages for negotiating cluster joins and leaves (JOIN/LEAVE request and response, respectively). In combination, these messages implement a coordinated switching (roaming) of CMs between clusters, which is discussed as part of further concepts in Section 4.4.
**Phase 5—CH Channel Selection:** In this phase, the CHs select their cluster channels for the secondary interface. This is done by sequentially picking items from a predefined pool of pairwise non-overlapping channels in the 2.4 and 5 GHz frequency bands. For the smallest channel bandwidth of 20 MHz, this results in up to three orthogonal channels in the 2.4 GHz band and 19 channels in the 5 GHz band according to European regulations. Despite higher achievable link data rates of the optional channel bandwidths of 40, 80, and 160 MHz introduced with 802.11n and 802.11ac, these are deliberately avoided in the CHaChA concept. The use of a large number of narrow channels instead of fewer wide channels facilitates the formation of clusters with independent collision domains, whose data transmissions can take place in parallel, especially with regard to densely populated mesh topologies [12].
  In practice, technical and regulatory restrictions may reduce the number of usable channels. In this context, the transmit filters of real WLAN transceivers are generally not able to completely limit the generated signal power to the respective channel bandwidth (e.g., 20 MHz). Therefore, despite maintaining a center frequency spacing of one channel bandwidth, depending on the intensity of the signal power, so-called Adjacent Channel Interference (ACI) between neighboring cells may occur [37,38]. To effectively avoid this, their frequency separation must be increased or the transmit power reduced. In addition, parts of the 5 GHz band are shared with radar technology and WLAN devices are required to detect and avoid radar signals, which might be intolerable for certain application scenarios. Similarly, there may be differences in frequency band support between different WLAN devices that must be considered when choosing a cluster channel. In this work, we specify the channel pool under the simplifying assumption of a homogeneous network of nodes with the same hardware capabilities.
  The CHaChA channel selection sequence is started by the MCH, claiming the first channel from the pool. It then adds the value pair of its own MAC address and chosen channel to the payload of a CHAN_SEL unicast message and sends it to the closest CH in terms of ALM path cost. This process is repeated by each subsequent CH until all CHs have claimed a channel. If the channel pool is exhausted before reaching the last CH of the sequence, channels are used multiple times following a classical greedy approach. To avoid interference between neighboring clusters, a CH reuses the channel of the CH in largest ALM distance. The last CH in the sequence sends a final message containing all MAC address/channel value pairs back to the MCH, which now triggers the transition to phase 6 via PHASE_6 messages. Figure 4e shows the intermediate result at the end of phase 5 in the example network.
  In the current concept, channel selection is based solely on the predefined channel pool, and no external WLAN cells are considered yet. In practice, CHs could also perform an active search for surrounding networks using 802.11 probe-request messages and preferentially select orthogonal channels. In addition, the concept so far focuses on channel selection for secondary interfaces that are used for communication within the mesh network. However, the allocation of additional free interfaces as Access Points (APs) to connect conventional WLAN clients without mesh capability could be done in a similar way. In the simplest case, the selected cluster channels could be shared by a virtual AP interface that is additionally created on the physical secondary interface. Alternatively, based on knowledge of current cluster channels and distance to other clusters, orthogonal channels could preferably be chosen for additional physical AP interfaces.
  With the end of phase 5, the periodically transmitted CH broadcast messages contain the cluster channel and the list of CMs associated with the cluster, in addition to the cluster’s mesh ID. The channel information is needed by CMs to configure their secondary interfaces. In turn, the number and constellation of participants are possible criteria for later cluster balancing. Thus, CMs may preferentially move to underpopulated neighboring clusters in the course of roaming activities (see Section 4.4.4).
**Phase 6—Cluster Interface Configuration:** In phase 6, all CMs configure their secondary WLAN interface according to the parameters announced for their respective cluster, enabling communication on the cluster channel. Figure 4f shows the final result after phase 6 in the example network. After configuring their secondary interfaces, all nodes switch directly to phase 7. As on the primary interface, the CHs now activate the proactive HWMP mode on the secondary interface. Unlike on the base channel, however, here they use the mode variant “proactive PREQ with PREP”, which causes bidirectional path creation. As a result, path information is maintained by CMs and their CH, and node failures can be mutually detected by checking the presence of path entries.
**Phase 7—Distributed Cluster Operation:** When reaching phase 7, the clustering procedure is complete. Network services and applications that can be appropriately mapped to different clusters benefit from higher performance as they communicate in parallel on orthogonal channels and in smaller broadcast domains, compared to the base channel. At the same time, unrestrained connectivity between the clusters is always guaranteed via the primary interface. By assigning applications to independent clusters, the load on the base channel can also be reduced, creating additional capacity for inter-cluster data exchange.
  At the application level, it should be noted that the primary and secondary interfaces of the mesh nodes operate in separate 802.11s networks, configured as different logical subnets with own IP address ranges. The current concept of CHaChA does not dictate how the configuration of these address ranges and the binding of applications should take place. In our evaluation in Section 6, all mesh interfaces were assigned fixed IP addresses, and we assumed that applications have the required prior knowledge. In practice, however, numerous strategies are conceivable. For example, automatically configured link-local IPv6 addresses of the primary and secondary interfaces could be used, which CHaChA provides to other applications as part of the knowledge about the cluster membership of nodes. Alternatively, CHs could take on extended DHCP and DNS functionality to implement an appropriate addressing and naming scheme for the various subnets.
**Handling errors in the phase sequence:** Possible errors in the phase sequence can be detected independently by the distributed nodes. For example, in the competition for MCH in phase 0, there is a theoretical risk of obtaining multiple MCHs as a result of continuous losses of CENT broadcast messages, especially if the parameters CENT_PERIOD and CENT_THRESH are chosen too small. However, with appropriate parameterization, this risk can be minimized. Based on the reception of MCH control messages of different sender addresses, a possible error case can be easily detected by each node. Subsequent problems can be detected by an exceeded (PHASE_X_TIMEOUT) within the phases. This could be caused by MCH failure or node disconnection, resulting in phase transitions not being initiated in time or not being received in parts of the network. Similarly, the channel selection sequence in phase 5 could be interrupted as a result of CH failure, so that a transition to phase 6 also does not occur.
  For error handling, nodes jump back to phase 0 as CFNs. If the reason was only a temporary disconnection to the MCH without affecting the phase sequence in the rest of the network, nodes can subsequently join one of the resulting clusters. On the other hand, if the MCH fails (or is permanently isolated), a new clustering is automatically started in the affected parts of the network. These and other mechanisms for handling failures and network changes are discussed in Section 4.4.5.

### 4.4. Online Cluster Adaptation

**Figure 5:**
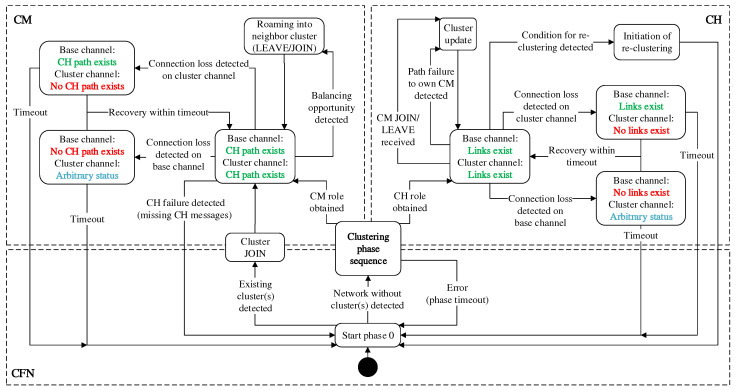
State diagram of the dynamic cluster adaptation mechanisms for long-term network operation (CFN: Cluster-Free Node, CM: Cluster Member, CH: Cluster Head). Reprinted from ref. [18].

Following the initial cluster formation, further concepts are required to react to changes in the network structure and to make cluster adjustments at runtime. With the exception of functions for joining an existing cluster constellation, which are performed in phase 0, all mechanisms take effect after completion of the clustering phase sequence in phase 7. The detection of connectivity problems and topology changes relies on CHaChA protocol messages at the application layer on the one hand, and on link and path information at the 802.11s link layer on the other hand. Figure 5 shows an overview of all handled cases as a state diagram.

#### 4.4.1. Joining a Clustered Network

For the initial cluster formation during network commissioning, as described in Section 4.3, a synchronous start of execution of CHaChA on all mesh nodes is assumed. However, in the practical operation of an urban backbone WMN, post-installation or relocation of devices is also to be expected. Further mechanisms are therefore used to subsequently incorporate nodes without having to perform a new initial cluster formation. They are defined as an extension of phase 0 of the CHaChA phase sequence (see entry point of the state diagram in Figure 5).

Provided that at least one active mesh link exists at the beginning of phase 0, a CFN first checks over the time duration {CH_THRESH · CH_PERIOD} if CH broadcast messages with complete cluster information (mesh ID and channel for the secondary interface) are sent on the base channel. These indicate that an initial cluster formation has already been performed, and the CFN has arrived subsequently. Alternatively, the receipt of MCH phase announcements signals a clustering that is still in progress and needs to finish first. For cluster joining, the CFN then sends a JOIN message to the CH with smallest ALM cost. Again, CHs in one-hop proximity are preferentially chosen. After configuring its secondary interface according to the cluster information (mesh ID and channel) announced by the CH, the CFN becomes a CM and switches directly to phase 7.

If there are neither CH messages nor phase announcements in phase 0, CFNs proceed with the normal phase sequence for initial cluster formation. If errors occur during the phases, detected by exceeding a timeout due to missing MCH phase announcements, nodes jump back to phase 0. Errors and connectivity problems that occur after successful clustering are also handled by jumping back to phase 0, as described in detail below. There, nodes attempt reintegration into the network or alternatively start a new clustering phase sequence.

#### 4.4.2. Node Isolation and Fault Detection

After successful completion of the clustering phase flow, nodes are either in the CM or CH role (see state diagram in Figure 5). By checking the status of their mesh interfaces, CMs and CHs can detect their own isolation as well as mutual disconnects. Possible causes for this are manifold and include, for example, dynamic obstacles, weather effects, relocation of devices in the course of network maintenance, or even device failures due to hardware defects or lack of power supply.

In addition, CMs may not be able to reach their CH through the secondary interface after initial clustering. Cluster joining in phase 4 already follows the strategy to select the closest CH in terms of ALM. However, due to the simultaneous and individual decision-making of all nodes, it is not guaranteed that they always form coherent groups. Therefore, after configuring the secondary interfaces of the nodes (setting the channel and mesh ID in phase 6), connectivity problems may occur within the clusters. Although the primary interface, statically configured to the common base channel, guarantees the highest possible connectivity, isolation on the cluster channel must still be handled by the mechanisms described below.

In all the situations mentioned, there is a loss of link and path information at the 802.11s MAC layer. Here, different constellations exist for the status of the primary interface on the base channel and the secondary interface on the cluster channel, which are handled differently depending on the node role. In Figure 5, the interface condition is highlighted in the corresponding states (green—no connection problems, red—connection failure, blue—arbitrary status).

**CM-side handling:** From the perspective of a CM, there are no connectivity problems if it reaches its CH as the anchor point of the cluster via both interfaces. This is guaranteed as long as 802.11s path information to the CH is available, while also assuming that at least one link exists per mesh interface. Since CHs use the proactive HWMP mode both on the base channel and within their cluster, path information to them is periodically updated on all nodes. In the state without connectivity problems, there is the possibility of coordinated switching (so-called roaming) between neighboring clusters with the goal of cluster balancing. These mechanisms are described in detail in Section 4.4.3 and Section 4.4.4.
  If a CM permanently loses the connection to its CH on the cluster or base channel, detected by exceeding a timeout (CONN_TIMEOUT) in the state without path information to the CH, it jumps back to phase 0 as CFN. Here, the mechanisms for reintegration into the network or repeated cluster formation take effect (see Section 4.4.1). Even with a mesh connection in place, CMs can detect a functional failure of their CH by missing CH broadcast messages at the application layer. CHs send these messages as periodic heartbeats and to announce their cluster information. Absence of the messages signals the loss of the CH role, e.g., as a result of software failures above the WLAN MAC layer. In this case, the affected CMs also switch back to phase 0 as CFNs.
**CH-side handling:** A CH makes the assumption of error-free operation as long as both of its mesh interfaces are connected. In this state, at least one link each exists to the base and cluster channels, and the CH’s main task is to respond to possible changes in its cluster. Join and leave requests of CMs, e.g., as a result of installing new nodes or in the course of roaming, require an update of the cluster information, which the CH announces by means of CH messages in the network. Similarly, the failure of an associated CM is detected if no path information is available to it, despite the proactive HWMP mode on the cluster channel.
  A CH detects its isolation by exceeding a timeout (CONN_TIMEOUT) in the state without link information. Analogous to the CM-side isolation detection, two possible situations are distinguished on the CH side: In the first case, there is no link at all on the primary interface, which is relevant for CHaChA coordination. Here, the state of the secondary interface is irrelevant. The second case, which affects only the secondary interface on the cluster channel, may arise for various reasons. On the one hand, CMs may fail so that links to nodes of neighboring clusters exist exclusively on the base channel. On the other hand, CMs may become disconnected from their CH due to the lack of intermediate nodes on the cluster channel, so that they too are only accessible via nodes of other clusters on the base channel. Since the CH loses its suitability as a coordinating reference node in each case, the situation must be handled accordingly. For this purpose, the CH jumps back to phase 0 as CFN. Here, the attempt is to reintegrate into the network, as described in Section 4.4.1, or alternatively, a new cluster formation takes place. In turn, the disconnection is also handled by the CMs of the cluster. They detect the loss of path information to the CH and also attempt to switch to a new cluster in phase 0.
  Moreover, independent of the isolation and failure handling, the CH-side initiation of a new cluster formation is conceivable. In Section 4.4.5, scenarios and conditions that could motivate such a re-clustering are discussed.

#### 4.4.3. Roaming between Clusters

The coordinated transition of a CM from its previous cluster to a neighboring cluster is referred to as roaming. After reaching phase 7, roaming operations are used for cluster balancing, which is explained in more detail in Section 4.4.4. Our concept follows basic ideas of the work JRCAP [11], which also achieves balanced clusters through roaming. In JRCAP, a change of cluster membership is reserved for so-called relay nodes alone. These are edge nodes that connect neighboring clusters. When changing clusters, a negotiation takes place between the relay node and the new or old CH using ATTACH and DETACH messages, which must be acknowledged by the CHs, respectively. This provides CH-side control of roaming operations. However, unlike CHaChA, JRCAP does not provide for a dedicated base channel on which CHs announce their cluster status throughout the network. For example, the size of neighboring clusters is known only to relay nodes, which must pass it on to CHs as part of the negotiation process. Instead, CHaChA proposes a roaming variant that relies on information from CH broadcast messages as well as local 802.11s connection information.

**Figure 6:**
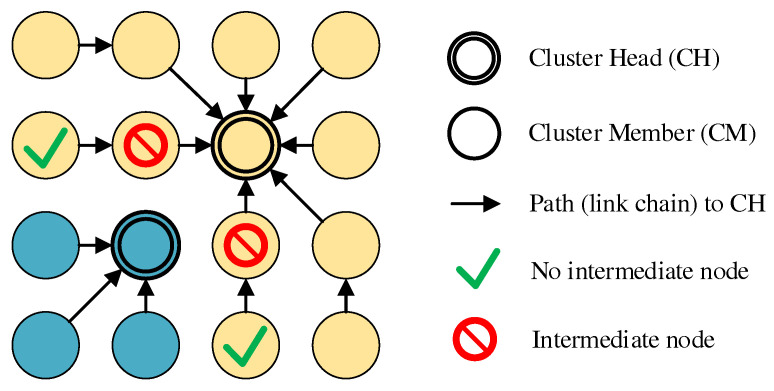
Roaming restrictions in an example network. Reprinted from ref. [18].

As in the related work JRCAP [11], the prerequisite for a CM to change clusters is that it is an edge node of its cluster. In addition, connection to at least one neighboring cluster must exist. CMs validate this using their 802.11s link and path lists as well as cluster information sent periodically by all CHs as broadcast messages on the base channel. This includes, among other things, the list of MAC addresses of all CMs of the respective cluster. If a mesh path exists to the CH of another cluster and at least one CM of this cluster is among the own mesh neighbors on the base channel, it is a neighboring cluster that can be switched to. If several possible roaming destinations exist, analogous to conventional cluster joining in CHaChA, the CH in smallest ALM distance is chosen. Another requirement is that the CM intending to roam does not itself act as intermediate node, connecting its CH and other CMs on the cluster channel. This is a conservative constraint, which ignores that alternative paths might exist via other nodes. However, it prevents clusters from being torn apart as a result of roaming, leading to undesirable isolation. To achieve this, neighboring CMs exchange so-called Next-Hop-to-CH (NH2CH) messages with each other from phase 7 onwards (see also Table 4 in Section 4.2). These messages provide information about which neighbor node (“next hop”) the sender of the message is currently using on the path to its CH. If a CM does not act as next hop for any of its neighbors, it may perform a cluster switch. Figure 6 illustrates the restrictions with an example in which, out of four edge nodes, only the two outer ones do not act as next hops and are therefore allowed to roam.

**Figure 7:**
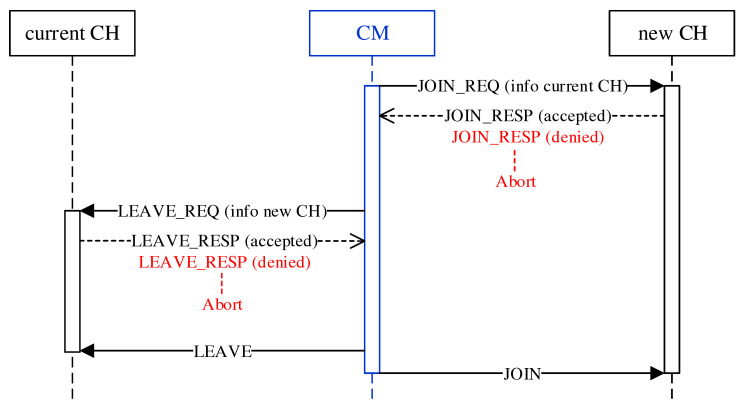
Sequence diagram for CM roaming between clusters. Reprinted from ref. [18].

The roaming procedure of a CM takes place as a handover between previous and new CH according to Figure 7. Since CMs make the decision to roam independently and possibly simultaneously, there is a risk of erratic cluster changes and oscillation behavior. In the course of a bidirectional negotiation (handshake), CHs therefore decide on the acceptance or rejection of roaming requests. For example, by CH-side specification of a minimum time interval between roaming operations, a restriction on the frequency of cluster switches can be realized. To join the new cluster, the CM first sends a request to its CH in the form of a JOIN_REQ unicast message. The CH of the new cluster responds with a JOIN_RESP message to confirm or deny the request. In the absence of a response or an explicit rejection, the CM terminates the roaming operation. On acceptance, the CM analogously sends a LEAVE_REQ message to its current CH, which acknowledges it with a LEAVE_RESP message. With a final LEAVE message, the CM leaves the previous cluster and joins the new cluster using a JOIN message. At the same time, it adopts the mesh configuration of the new cluster (mesh ID and channel) on its secondary interface. Possible disconnections between CM and CH could cause non-delivery of the final LEAVE/JOIN messages. However, such a situation would be handled subsequently by the isolation/failure detection.

The JOIN_REQ and LEAVE_REQ message carry the MAC address of the source and destination cluster’s CH, respectively, to enable the mutual decision of accepting or rejecting CMs. The cluster membership of CMs can in principle also be obtained from the information in the periodic CH announcement messages. However, these are sent on the base channel as unreliable broadcasts using UDP. In contrast, roaming negotiation is done via reliable unicast messages based on TCP. This ensures the availability of up-to-date information.

#### 4.4.4. Cluster Balancing

The balancing of a cluster constellation can be done according to different aspects [39]. If the focus is on channel utilization, for example, nodes that communicate frequently could be grouped into smaller clusters, while nodes that communicate infrequently could be grouped into larger clusters. The logical coherence of devices at the application layer could also be taken into account. However, this requires prior knowledge of the type and communication patterns of applications or active observation of channel usage. On the other hand, if spatial node density is used as criterion, many small clusters on non-overlapping channels might be desirable in densely populated regions to reduce co-channel interference. In sparsely populated regions, again, larger clusters would be preferred to guarantee redundant paths and prevent node isolation. Such strategies, however, require knowledge of the spatial distribution of nodes or estimation of their positions.

An intuitive approach that needs only little information is balancing the number of cluster participants [11]. With regard to network management scenarios, this pursues the goal of distributing the communication and management effort evenly among the clusters on the one hand. On the other hand, this limits the damage caused by a CH failure, as a result of which affected CMs have to reintegrate into the cluster constellation. The clustering phase sequence described in Section 4.3 does not guarantee the emergence of clusters of equal size. Due to the individual and simultaneous decision-making of all nodes during initial cluster joining, an imbalance in cluster sizes can only be detected afterwards. Therefore, the balancing concept presented below only takes effect after the clustering phase sequence is completed in phase 7. As in the work JRCAP [11], balancing of adjacent clusters is also proposed in CHaChA. The measure for balancing is the roaming of CMs at the edge between neighboring clusters previously described in Section 4.4.3.

If an edge CM is eligible for roaming, it checks the need for cluster balancing after receiving new CH broadcast messages for its neighbor clusters, which contain the list of CMs of the respective cluster. After determining the current cluster sizes from the information in the CH messages, the CM compares its own cluster size with that of its neighboring clusters. If any of these clusters comprise at least two nodes below its own cluster size, the CM switches to the closest neighboring cluster according to its CH path cost (ALM). Thereby, a roaming direction from larger to smaller clusters is achieved.

**Figure 8:**
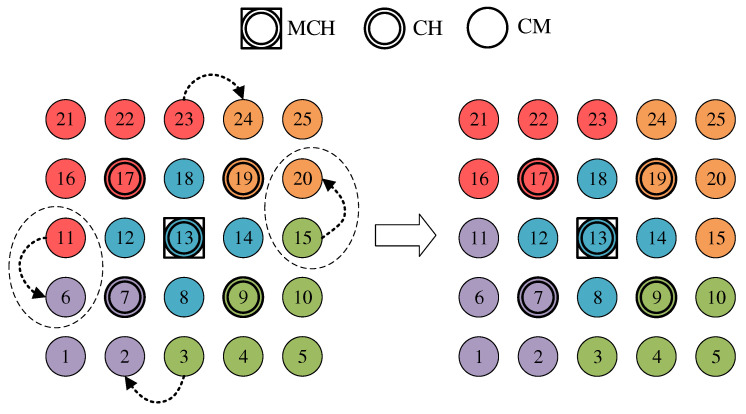
Cluster balancing in an example network (encircled roaming opportunities are detected first and are therefore executed). Reprinted from ref. [18].

Figure 8 shows the balancing using the example of a 5 × 5-node grid topology with five clusters. In the unbalanced initial state (Figure 8 left), the red and green clusters have a number of participants above the targeted average size of five nodes. The edge nodes 3 and 15 (green cluster) and 11 and 23 (red cluster) each possess the ability to roam into the neighboring cluster that is smaller by two nodes, with the simplified assumption that nodes 11 and 15 recognize this fact earlier and perform roaming. The result is a balanced cluster constellation (Figure 8 right).

To coordinate roaming operations, each CH maintains a list of neighboring clusters to which CMs have recently migrated or from which CMs have joined in return. After each roaming operation, further transitions to the same destination cluster or from the same origin cluster are temporarily rejected to reduce conflicting roaming operations of CMs that have detected a balancing opportunity simultaneously. This strategy is a compromise between quickly balancing widely varying cluster sizes and preventing avalanche effects with the risk of oscillating clusters.

#### 4.4.5. Re-Clustering

Based on the concepts for subsequent cluster joining, isolation and failure detection, and cluster balancing, it is possible to respond to topology changes and connectivity problems even after the initial clustering phase has been completed. For example, subsequently installed devices can automatically integrate into an existing network, which is capable of independently balancing a resulting imbalance in cluster sizes. Similarly, individual CH failures can be compensated by moving the associated CMs to surrounding clusters.

Beyond that, however, scenarios are conceivable that require a revision of the overall cluster constellation. One possible reason are severe topology changes that might be triggered, e.g., by the addition of a large number of nodes, frequent CH failures, or isolation of larger sub-networks. There could also be a persistent imbalance in cluster sizes that cannot be addressed by roaming due to an absence of cluster edge nodes.

Distributed, asynchronous problem handling by individual nodes may not always be able to produce a cluster constellation suitable for the particular use case. A more reliable alternative here is the initiation of re-clustering. In CHaChA, detecting the need for re-clustering as well as its coordinated announcement in the network are conceptually reserved for CHs. Possible detection criteria are the evolution of the total number of nodes (network size *N*) or the CM/CH ratio since the last cluster formation.

No re-clustering mechanisms have yet been implemented and tested in the context of this work, but they are feasible based on the previously described concepts. After detecting a condition for re-clustering, CHs take over its initiation on the base channel (primary interface). An intuitive approach is to send PHASE_0 broadcast messages, analogous to the control of phase transitions by the MCH in the clustering phase sequence. This causes a coordinated switching of all nodes into phase 0, whereupon re-clustering is performed. Even during re-clustering, connectivity in the network is always maintained via the statically configured primary interface on the base channel.

## 5. Prototype Implementation

**Figure 9:**
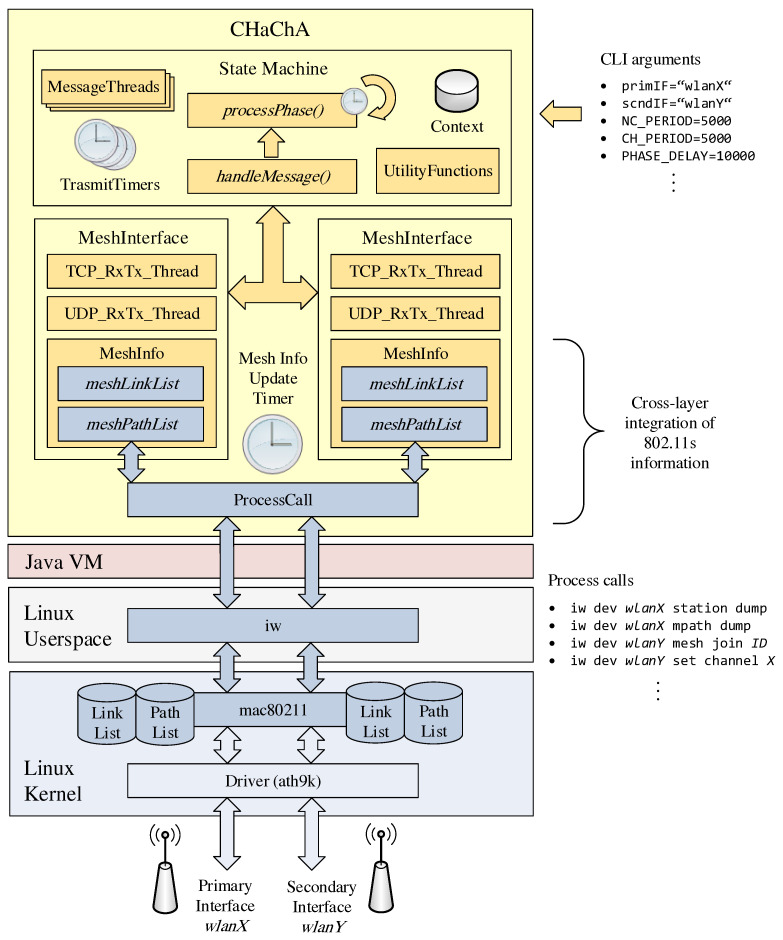
Software architecture of the CHaChA prototype implementation. Reprinted from ref. [18].

The concepts of our clustering approach CHaChA were realized as a Java application. Figure 9 shows the simplified architecture of the resulting software prototype. The implementation runs on Linux operating systems with 802.11s support. Configuration options (e.g., device names of the WLAN interfaces) as well as any parameters of the CHaChA algorithm (time constants and thresholds, see Section 4.2) can be passed individually as arguments at program start.

The phase sequence for initial clustering, including the mechanisms for online cluster adaptation that take effect after reaching phase 7, was implemented as a state machine according to the concepts described in Section 4.3 and Section 4.4. Depending on the phase and node role, state transitions depend on timeouts on the one hand and incoming CHaChA protocol messages on the other. The periodic sending of different message types, such as CENT messages for MCH election, MCH phase announcements, or CH info broadcasts is done in separate threads with their own transmission timers, which are active depending on the phase. Other functions realize the calculation of the various CHaChA metrics (see Section 4.2) as well as decisions based on the connection status on the base and cluster channels.

For TCP/UDP communication and connection information management, the program maintains Java objects that represent the node’s mesh interfaces. A dedicated update thread handles the cyclic retrieval of the link and path lists from the Linux kernel and thus realizes a cross-layer integration of the 802.11s information into the application layer. The access to the 802.11s data structures of the Linux kernel module mac80211 is done by process calls of the command line program iw [40]. Here, the text outputs of the link and path lists read from iw are converted into corresponding Java data types (String lists) and stored within the MeshInterface objects. In addition to the connection information, each interface has a separate thread for sending and receiving CHaChA protocol messages via TCP and UDP, respectively. While TCP is used for reliable unicast transmissions to selected nodes and thus also for messages whose range should be limited to their own mesh neighbors, UDP is currently used exclusively for sending network-wide announcements as broadcast messages.

The structure of the CHaChA protocol messages is summarized in Table 6, where the payload of an example message is given for each message type (opcode). For the prototypical implementation, a plaintext encoding was chosen, which allowed a simple analysis of the packet captures during the evaluation. To this end, all payload components, including MAC addresses, metric numeric values, and WLAN channel numbers, are serialized as Java String representations when sent and converted back to their corresponding data types at the receiving end. Starting with the opcode of the message, the fields of the payload are delimited by a reserved delimiter character (‘|’) and can thus be distinguished on reception. The simplest message types PHASE_X, PCH, and JOIN_REQ/LEAVE_REQ consist only of their opcode. The JOIN_RESP/LEAVE_RESP response messages additionally contain a 1 or 0 as an indication of acceptance or rejection of roaming requests. Similarly, the CENT, NC, and WNPR messages carry the numeric value of their metric, while the NH2CH message contains the MAC address of the neighboring node via which the mesh path to its own CH is established. The more complex CH announcement messages consist of the cluster’s mesh ID and channel number as well as the list of CM MAC addresses, in addition to their opcode. The CHAN_SEL message can also reach a certain size depending on the amount of CHs, as it is gradually extended by pairs of CH MAC address and cluster channel during the channel selection sequence in phase 5. In practice, more compact encodings, such as the use of binary representations for opcodes and other numerical values and the definition of fixed field lengths without separators, would allow for much smaller absolute message sizes. Similarly, compression methods could be used, such as run-length coding or DEFLATE-based methods [41]. However, as part of the evaluation of the clustering communication costs in Section 6.2.4, the focus was on examining the relative relationship between network size and the amount of data sent.

## 6. Experimental Validation

### 6.1. Testbed Setup

**Figure 10:**
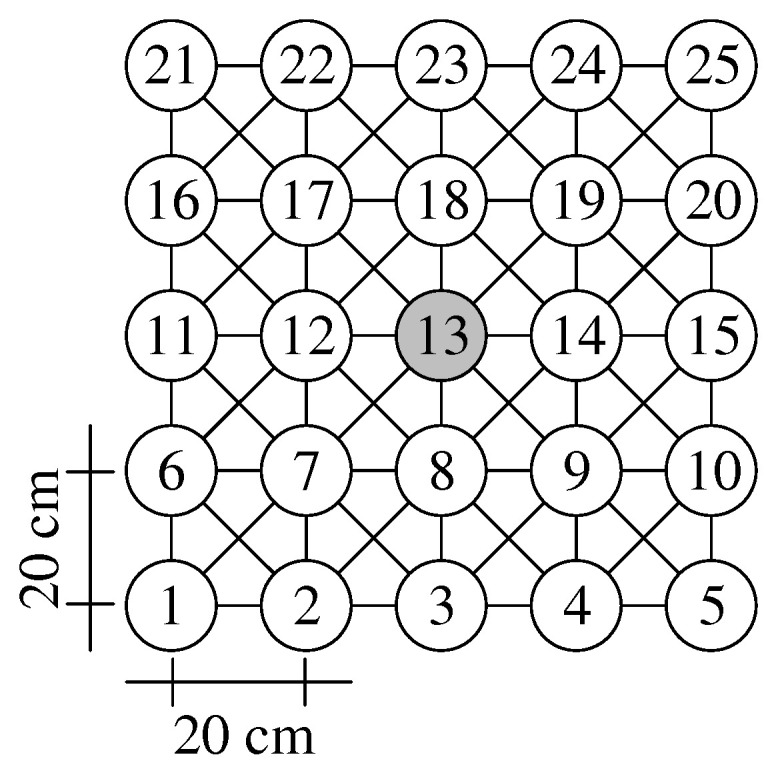
Testbed grid setup and node numbering.

We evaluated our CHaChA prototype in a practical testbed, comprising 25 Intel Galileo single-board computers. The testbed is arranged in a regular 5 × 5-node grid setup, offering a network diameter of four hops and a symmetric center position (node 13). Figure 10 shows the grid geometry and node numbering. Based on our miniaturization approach Mini-Mesh [42], using RF attenuators and a fixed 802.11 data rate and TX power combination, we achieve a substantial reduction of transmission range. As a result, only grid neighbors communicate directly, whereas multi-hop paths are established between more faraway nodes by the 802.11s routing protocol HWMP. This allows us to create indoor multi-hop setups in a scale of approximately 1:560.

In [42], we introduced this scaling approach and validated its applicability via comparative measurements in outdoor setups without RF attenuators. The miniaturized 5 × 5-node grid topology in our lab corresponds to an un-miniaturized wireless mesh backbone covering an area of more than 300,000 m${}^{2}$. The used topology mimics, e.g., a city-district mesh network where backbone nodes are installed on rooftops or streetlight poles in a grid-like manner. The regular layout of such a grid topology also simplifies expectations in terms of node neighborhoods and resulting cluster constellations.

Our device configuration is given in Table 7. Besides an on-board Ethernet interface, attached to a wired control network for test automation, each node is equipped with a dual-antenna 802.11n mPCIe card. All cards operate at a fixed 802.11 data rate and start on a default channel, not used otherwise in our institute building. In [42], we conducted a general performance assessment of our device platform (Intel Galileo Gen. 1, 400 MHz single-core CPU) for the 802.11n HT data rates MCS 0–15 (i.e., from BPSK 1/2 to 64-QAM 5/6). Generating saturated TCP/UDP traffic with MTU-sized data frames, MCS 3 was determined as the highest data rate that could be fully utilized before our devices ran into CPU performance limitations. Being the highest rate sensibly applicable in our current setup, MCS 3 was therefore also chosen for the evaluation of CHaChA.

The concept of CHaChA mandates the presence of two WLAN interfaces per node to be able to communicate simultaneously on the base and cluster channels after initial cluster formation. In general, it would be possible to equip each Galileo board with a USB WLAN dongle in addition to its mPCIe WLAN card. However, no USB products are available that match the chipset of the mPCIe cards (Atheros AR9280) while supporting external antennas for attaching attenuators. We deliberately avoid a mixed hardware configuration of WLAN interfaces with different range and performance characteristics and instead only use the mPCIe card as primary interface in each test case. It is configured to the fixed WLAN channel 149 (center frequency $f_{c}=$ 5745 MHz), serving as base channel. For the investigation of the initial cluster formation (see Section 6.2), a second interface is not necessary, as all CHaChA control messages are sent on the base channel, and there is the possibility to select the cluster channel only virtually without actually performing the configuration of the secondary interface. Accordingly, when testing dynamic cluster adaptation (see Section 6.3), only topology changes on the base channel are examined.

### 6.2. Clustering in Static Topologies

In the following, CHaChA is investigated in static mesh topologies of different sizes in terms of clustering result, duration, and message overhead. First, Section 6.2.1 describes the estimation of the clustering duration depending on the parameters of the algorithm. After Section 6.2.2 explains the general experiment procedure, Section 6.2.3 discusses the clustering of a 5 × 5-node grid topology for a conservative parameterization of CHaChA. Here, the focus is on confirming the reproducibility of the results as well as the previously estimated clustering duration. Subsequently, in Section 6.2.4, several grid topologies of increasing node count are examined for a second parameterization to determine the impact of network size on clustering duration and message overhead.

#### 6.2.1. Estimation of Clustering Duration

Designing the initial cluster formation as a phase sequence also brings benefits for its practical analyzability. For example, the time required for the phase sequence can be estimated in advance, since it is largely determined by the parameterization of the algorithm. Basically, the parameters are chosen as a compromise between low overhead (clustering duration and number of control messages sent) and required robustness (high delivery probability of the information required for clustering). In practice, the parameterization depends, for example, on the expected network size and link quality as well as the processing speed of the mesh nodes. For a detailed description of all CHaChA parameters, we refer to Section 4.2, Table 5.

**Figure 11:**
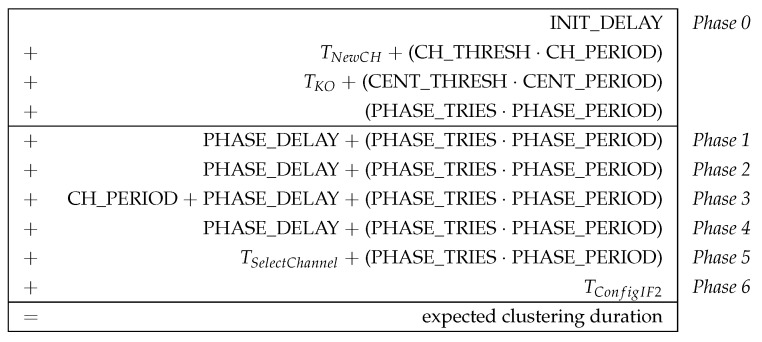
Time components for estimating the clustering duration. Reprinted from ref. [18].

The clustering duration results, to a large part, from fixed waiting times before phase transitions as well as periodic retransmissions of broadcast messages, which mainly concern the phase announcements of the MCH. Figure 11 shows the general estimation of the clustering duration as a composition of the temporal contributions of all phases. Here, it is assumed that all nodes start executing the phase sequence simultaneously. In practical scenarios, we expect the clustering phase sequence to be triggered by network operators after initial node commissioning. Depending on the parametrization of the algorithm (i.e., granting up to several seconds “slack” per phase), only a loosely synchronized start of CHaChA on all nodes is required. Triggering a new clustering run will likely be done via the mesh network’s base channel, instead of a separate Ethernet control channel as in our testbed. Apart from that, also a loosely synchronized system time (e.g., achieved by using NTP on the base channel) would allow for planning the start of future clustering runs directly on the nodes.

**Figure 12:**
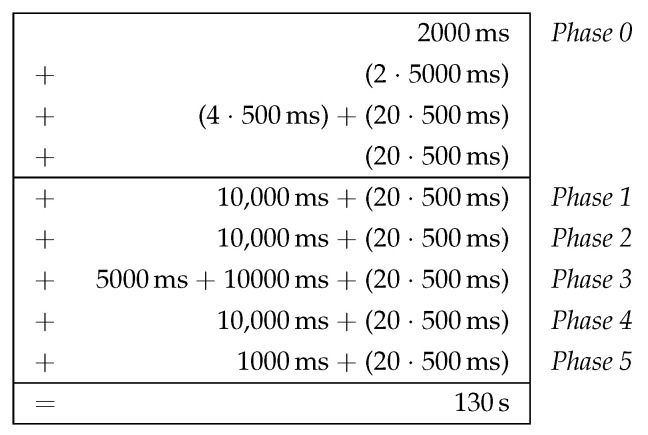
Estimated clustering duration for parameterization P1 (value given in Table 8). Reprinted from ref. [18].

Figure 12 shows a specific time estimation, applying CHaChA parameterization variant P1 (described later in Section 6.2.3) to the general estimation given in Figure 11. P1 served as initial parameterization for the practical assessment of the prototype (also see Table 8). It represents a generous choice of the time constants and thresholds to ensure robust clustering, resulting in an estimated duration of 130 s.

The time requirement in phase 0 (network entry and MCH election) is composed of four components. When the CHaChA prototype is started, it first waits a fixed time INIT_DELAY to create objects and data structures for the network interfaces and fill them with information from the operating system. This parameter is thus not part of the clustering algorithm, but depends on the implementation and hardware/software platform. Adjusted to the test environment, a value of 2 s was selected for INIT_DELAY. After its initialization, a node checks whether clusters already exist and the phase sequence can be skipped. To do this, it waits for CH_THRESH transmission periods of length CH_PERIOD for the reception of CH broadcast messages. If new messages of previously unknown CHs arrive before this time is exceeded, the timer is reset. The delay that can be caused by getting to know new CHs is summarized as $T_{NewCH}$. It depends not only on the time offset of the CH broadcasts, but also on their propagation time in the network. The clustering experiments described below were always started in a newly initialized network on the base channel, so $T_{NewCH}$ could be neglected.

If no CHs are available, MCH election is started. All nodes periodically announce their CENT metric via broadcast in the network at intervals of CENT_PERIOD. Upon receiving a higher CENT value, nodes are eliminated according to the K.O. principle. The node with highest metric wins the race after CENT_THRESH consecutive broadcasts without receiving a message in return. The convergence time of the K.O. method required for eliminating all nodes with lower metrics is summarized as $T_{KO}$ in Figure 11. It also depends on the broadcast propagation time for the respective network size/topology and is a multiple of the broadcast period CENT_PERIOD. Depending on the topology, the period should be chosen large enough to ensure complete broadcast propagation of all nodes within one round. If all nodes first perform a broadcast before receiving a message, at least two broadcast rounds are required for the K.O. procedure. Additional rounds are caused, e.g., by a too-small choice of CENT_PERIOD or the loss of broadcast messages in the network. In sample experiments using the 5 × 5-node grid topology and a broadcast period of 500 ms, convergence was observed after up to four broadcast rounds. As a simplification, $T_{KO}$ was subsequently estimated with a fixed length of (4 · CENT_PERIOD).

Following the selection of the MCH, it initiates the phase transition by means of periodic broadcast messages. This signaling procedure corresponds to the time requirement (PHASE_TRIES · PHASE_PERIOD). In our evaluation, the same repetition number and transmission period were used for all phase transitions. Thus, the respective duration of the subsequent phases 1–4 was composed of the same announcement duration plus a delay PHASE_DELAY. The MCH considers this delay prior to the initiation of the phase transition to ensure information exchange of all nodes including stabilization of metrics in each phase. In phase 3, an additional waiting time of CH_PERIOD (length of a CH broadcast period) is introduced to ensure robust cluster announcement independent of the PHASE_DELAY parameter.

Contrarily, announcement of the transition from phase 5 to phase 6 occurs directly after the channel selection conducted by the CHs and MCH. The time required for the channel selection sequence and its TCP unicast message exchange is summarized by $T_{SelectChannel}$. Besides the number of CHs, it also depends on the network topology (average distance between CHs) and transmission speed. Hence, as for $T_{KO}$, a general estimate of $T_{SelectChannel}$ is difficult. In sample experiments with the 5 × 5-node grid topology, where five CHs were created at a maximum distance of two hops from each other and the WLAN data rate setting MCH 3 (gross data rate of 26 Mbit/s) was applied, $T_{SelectChannel}$ stayed below 1 s. We further simplified its estimation using this upper value. The time $T_{ConfigIF2}$ theoretically required in phase 6 to configure the nodes’ secondary interfaces could be ignored in the experiments, since only the primary interfaces were used. In practice, $T_{ConfigIF2}$ depends on the device platform used, but usually the configuration of the WLAN channel including network joining requires only fractions of a second [43]. The subsequent transition from phase 6 to distributed cluster operation (“phase 7”) is always performed independently by all nodes, so there is no additional waiting time.

#### 6.2.2. Experiment Procedure

Depending on the topology under investigation, only the respective testbed nodes were active. The CHaChA prototype was started simultaneously on all devices via the Ethernet control network. At the beginning of each measurement, all nodes were already connected to each other on the base channel (single-channel mesh network). Clustering was considered complete once the last node reached phase 7 of the CHaChA phase sequence. As described in Section 6.1, no secondary interfaces were installed. All nodes applied the cluster channel selection and configuration in phases 5 and 6 only virtually and stored the result for subsequent analysis. Five channels (36, 40, 44, 48, 158) with no overlap to the base channel 149 ($f_{c}=$ 5745 MHz) were given as an exemplary cluster channel pool. For the topology sizes investigated in this work, with up to five clusters being created, this specification was sufficient. In practice, with a channel bandwidth of 20 MHz, up to 19 orthogonal channels are possible in the 5 GHz band [44]. As explained in Section 4.3, the concept of CHaChA also considers re-selection of already used channels by means of a distance heuristic.

In addition to a log file to record its actions in the phase sequence, each node produced a capture of its outgoing TCP and UDP messages using the command line tool tcpdump. At the end of each measurement, the CHaChA prototype also produced a separate status file that contained information about the time difference between program start and reaching phase 7, the node’s final role, and information about its cluster (CH and cluster channel). Any files generated on the nodes were stored in a RAM file system (Linux tmpfs), so that possible influences of slow SD card write operations were avoided.

Initialization of the mesh network and CHaChA instances, as well as automation of the experiments, were accomplished by command line scripts. These were run partly on the mesh nodes and partly on a laboratory PC connected to the test environment via the separate Ethernet control network. Following a series of measurements (series of clustering runs), the log/status files and tcpdump packet captures of all nodes were retrieved and processed. An analysis tool, developed as a student research project using the free MATLAB alternative Octave [45], was used to visualize all formed cluster constellations and to determine their respective occurrence rates in the measurement series. The grid positions of the nodes in the visualization were statically predefined. Based on each node’s status information (role and cluster membership), it was highlighted and colored accordingly. From the collected log/status files and packet captures, the tool also calculated the mean clustering duration and communication overhead generated by CHaChA control messages (TCP/UDP data size and message count).

#### 6.2.3. Assessment and Reproducibility Analysis

First, CHaChA was investigated in a test setup with 25 devices (5 × 5-node grid) with respect to clustering result, duration, and communication overhead. The focus was on functional testing of the clustering phase sequence and analyzing the reproducibility of its results under controlled laboratory conditions. The 5 × 5-node arrangement was deliberately chosen, representing the largest of the mesh topologies considered in this work. Table 8 shows the used parameterization variant P1 of the time constants and thresholds of the CHaChA algorithm. A generous choice of waiting times and broadcast retransmissions was initially intended to ensure robust clustering.

**Figure 13:**
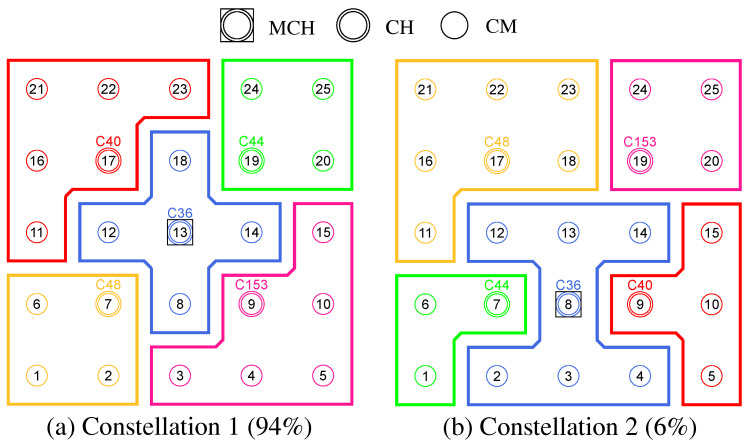
Cluster constellations in the 5 × 5-node grid for parameterization P1 (cluster channels (“C#”) highlighted in color; occurrence rate given in %). Reprinted with permission from ref. [17]. © 2018 IEEE.

The measurement series comprised 50 clustering runs and all results (time required and amount of data sent) were averaged. Figure 13 shows the cluster constellations created in the measurement series. The nodes belonging to a cluster are shown in the same color according to their cluster channel chosen in phase 5. The node roles MCH, CH, and CM are marked as indicated in the legend. As expected for the static test setup, reproducible clustering results were obtained. Overall, only two different constellations emerged. Moreover, constellation 1 (Figure 13a) emerged particularly frequently with an occurrence rate of 94%, i.e., in 47 out of 50 runs.

In the formation of this constellation, the centrally positioned node 13 was chosen as MCH in the competition of phase 0 due to its CENT metric. In phase 1, the nodes of the inner 3 × 3 grid obtained the PCH role because they each possessed a maximum NC metric with eight neighboring nodes. Among these nine PCHs, as expected, the four corner nodes 7, 9, 17, and 19 won the WNPR metric comparison in phase 2. Together with the MCH already chosen in phase 0, five nodes thus received the CH role. After network-wide CH announcement in phase 3, joining of the CMs in phase 4 based on their ALM cost to the CHs already resulted in largely balanced clusters. As shown in Figure 13a, the central cluster of the MCH reached a size of 5 nodes, consistent with the targeted average cluster size for 25 nodes and 5 clusters. The size of the surrounding clusters each differed from this by one node. The roaming mechanism described in Section 4.4.3 would lead to a balancing of cluster sizes after entering phase 7, with one edge node of the red and purple clusters each moving to the orange and green clusters, respectively. Channel selection in phase 5, starting at node 13 (MCH), was done in order of smallest ALM distance to each of the remaining CHs. Sequentially, nodes 13, 17, 19, 7, and 9 took channels 36, 40, 44, 48, and 158 from the predefined pool of orthogonal WLAN channels in the 5 GHz band. After virtual application of the channel configuration in phase 6, all nodes switched to phase 7, thereby completing the cluster formation.

The second cluster constellation (see Figure 13b) was formed in only 3 of 50 runs (occurrence rate of 6%). Its main difference from constellation 1 was that node 8 instead of node 13 won the MCH election. This was caused by deviations in the ALM cost between nodes during phase 0. The ALM is part of the calculation of the CENT metric on which the MCH election is based. The formation of different clusters was then caused by the shifted MCH position, as CMs preferentially join the cluster of the MCH if it is in their neighborhood. In scenarios with higher node count, network diameter, and dynamics, small deviations of the MCH position from the center are expected and acceptable in a heuristic approach.

In addition to the clustering result, the average duration and message overhead of the phase sequence were determined. For the generous choice of time constants and thresholds (see parameterization P1 in Table 8), applying the duration estimation according to Section 6.2.1 resulted in an expected time of 130 s. The mean duration measured in the experiments was 135.5 s (standard deviation 0.9 s), only slightly above the estimate. During this clustering period, an average of 9699 (standard deviation 42) TCP messages with a total data size of 1113.7 kB (standard deviation 4.9 kB) were sent, including all TCP ACK and handshake messages for connection setup and teardown, respectively. In addition, our prototype generated an average of 5248 (standard deviation 152) UDP messages with a total data size of 627.8 kB (standard deviation 20.4 kB). All results include the traffic generated by multi-hop message forwarding and any WLAN retransmissions.

The average total TCP/UDP traffic of 1740 kB, generated over a clustering duration of 135.5 s, can be simplified as overhead traffic with an effective data rate of circa 100 kbit/s. It should be noted that each node in the clustering phase sequence generates only a fraction of the total traffic, and thus this traffic is distributed across all links in the network. The higher message count and traffic share for TCP compared to UDP are on the one hand due to its inherently higher protocol overhead (longer header, acknowledged communication, three-way handshake). On the other hand, in our current prototype implementation, individual TCP connections are established and released for each reliable information exchange. A future optimization could be to keep the connections to neighboring nodes open, since TCP is mainly used to communicate with mesh peers (NC, PCH, and WNPR messages). In comparison, UDP-based broadcasts (CENT, CH, and PHASE messages), including their forwarding in the network, resulted in only slightly more than half the number of messages sent with TCP. This also corresponds to the amount of UDP data generated. However, the number of individual transmissions required to disseminate broadcast information is highly dependent on the network topology and error probability [46]. The UDP traffic share could therefore increase in larger networks.

In the CHaChA concept (see Section 4.2), several design decisions were made to keep communication overhead low even in larger mesh topologies. Metrics are exchanged using TCP unicast messages only between neighboring nodes and exclusively during the clustering phase sequence. After initial cluster formation in phase 7, mesh peers only exchange NH2CH messages, which serve to constrain roaming operations. Roaming of cluster edge nodes may result in occasional transmission of additional TCP messages (LEAVE_REQ/RESP or JOIN_REQ/RESP). In addition, network-wide UDP broadcasts are generated only by CHs, except for CENT messages during MCH election in phase 0. After the initial phase sequence, only CH broadcast messages for the announcement of cluster information are sent periodically. The detection of connection problems is possible on all nodes by merely checking local 802.11s link layer information.

#### 6.2.4. Results for Different Grid Sizes

Based on the results obtained with the conservative parameterization P1 in the 5 × 5-node grid, topologies of different grid sizes were investigated. In advance, the parameterization P2 given in Table 9 was determined experimentally in the 5 × 5-node grid. The objective was to reduce the clustering duration while maintaining the reproducibility of the previously obtained clustering results. Therefore, we gradually decreased parameters relevant to the duration and verified that no negative effects on the clustering result occurred. First, the time required in phase 0 was lowered. On the one hand, the initial check for already existing clusters was deactivated (CH_THRESH =0), since a synchronous execution of all devices was ensured in the experiments, and this fixed waiting time could thus be omitted in contrast to real scenarios. On the other hand, the threshold for consecutively received CENT messages was halved (CENT_THRESH =10), which reduced the time required by the K.O. competition for MCH election. Additionally, the delay before phase transitions (PHASE_X_DELAY) was reduced from 10 to 2 s. Likewise, the number of retransmissions (PHASE_X_TRIES) of the associated announcement messages was halved. Both parameters significantly determine the duration of the individual phases (see estimation in Section 6.2.1). Due to the shorter phase duration, the transmission intervals for NC and CH messages were reduced accordingly to ensure timely delivery within their respective phases.

With parameterization P2, the average clustering duration was reduced to just under 40% of the time required with parameterization P1 (55.6 s vs. 135.5 s). The previously most frequently generated cluster constellation (see Figure 13a) could still be reproduced in nine of ten measurements. The parameter values listed in Table 9 are specific to our testbed Mini-Mesh and its device platform [42]. It can be assumed that even lower times can be achieved with more powerful hardware.

In the following paragraphs, clustering results obtained with parameterization P2 for different network sizes are discussed. We considered grid topologies with 2 × 2 to 5 × 5 nodes. The practical procedure was unchanged from the experiments described in Section 6.2.2. However, for the trend analysis as main focus, we conducted only 10 measurements per topology and averaged their results. First, the generated cluster constellations are discussed and their formation is explained in the context of the algorithm. This is followed by a comparison of the scenarios with respect to clustering duration and communication overhead.

**Figure 14:**
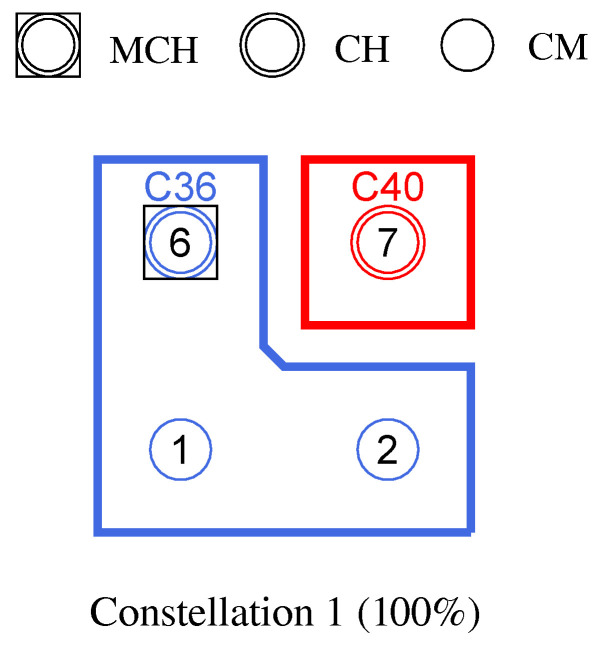
Cluster constellations in the 2 × 2-node grid for parameterization P2 (cluster channels (“C#”) highlighted in color; occurrence rate given in %). Reprinted from ref. [18].

**Figure 15:**
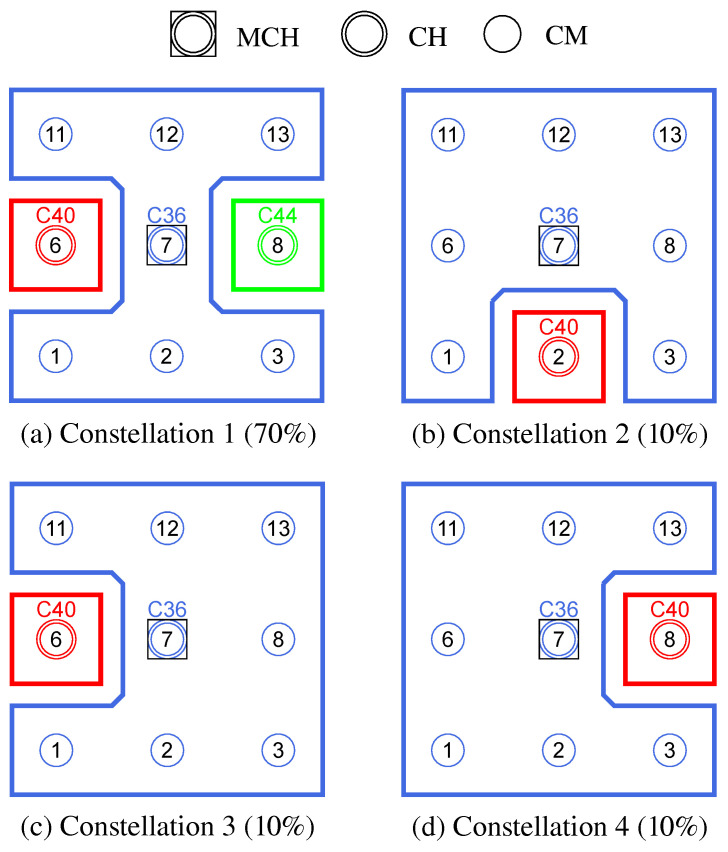
Cluster constellations in the 3 × 3-node grid for parameterization P2 (cluster channels (“C#”) highlighted in color; occurrence rate given in %). Reprinted from ref. [18].

**Figure 16:**
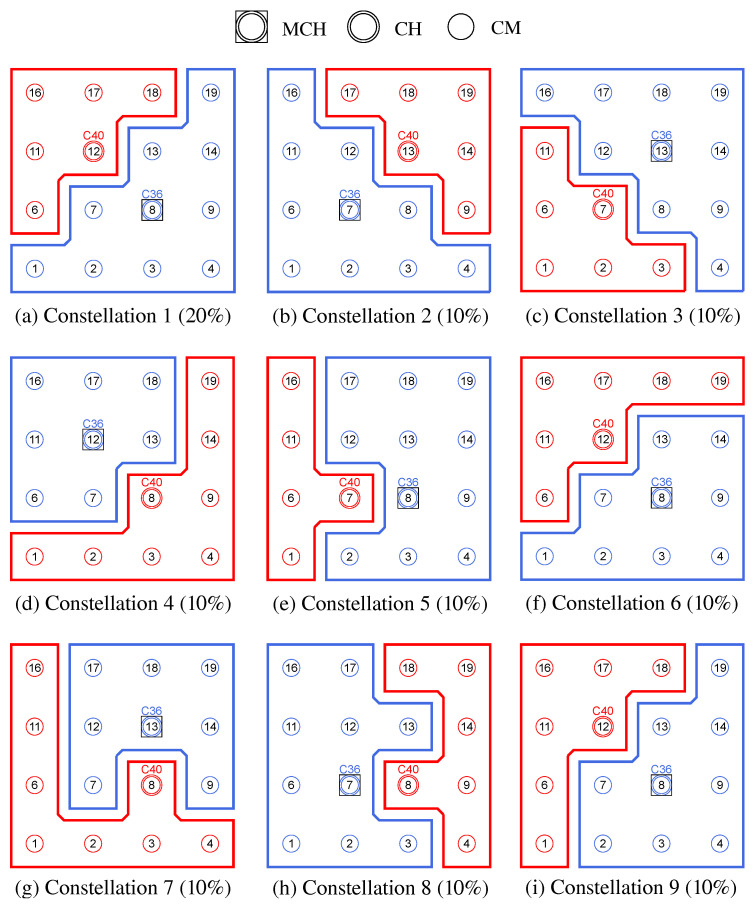
Cluster constellations in the 4 × 4-node grid for parameterization P2 (cluster channels (“C#”) highlighted in color; occurrence rate given in %). Reprinted from ref. [18].

**Figure 17:**
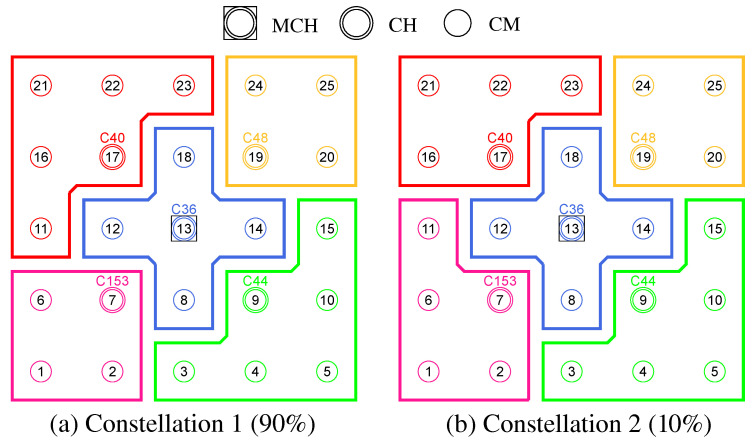
Cluster constellations in the 5 × 5-node grid for parameterization P2 (cluster channels (“C#”) highlighted in color; occurrence rate given in %). Reprinted from ref. [18].

**Figure 18:**
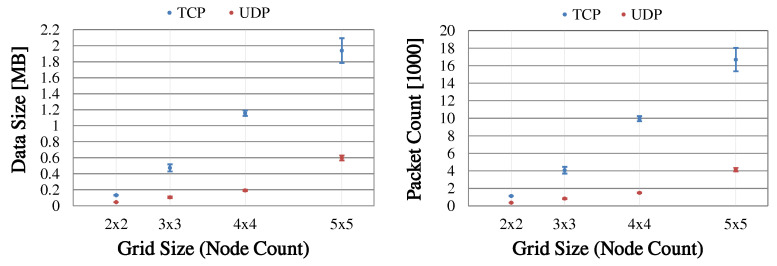
TCP/UDP overhead traffic data size (left figure) and packet count (right figure) depending on grid size/node count (parameterization P2; data points with standard deviation). Reprinted from ref. [18].

**Figure 19:**
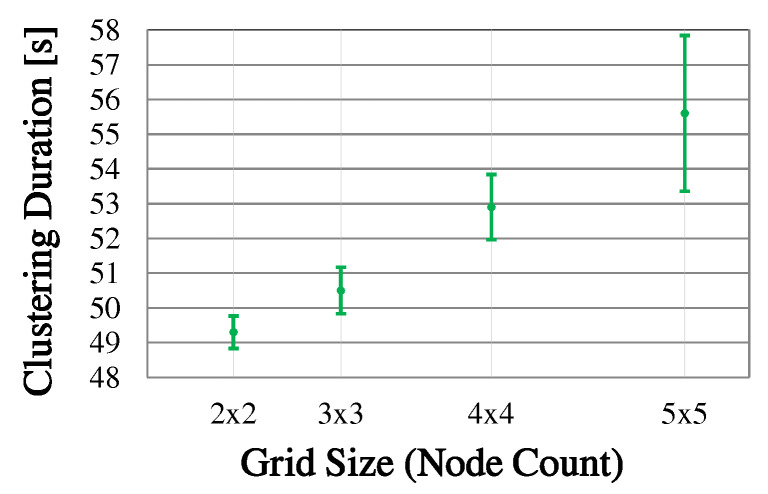
Clustering duration depending on grid size/node count (parameterization P2; data points with standard deviation). Reprinted from ref. [18].

**2 × 2-node grid:** In the smallest topology, the constellation with two clusters shown in Figure 14 was formed consistently in all measurements. Although no central node position existed in the 2 × 2 setup, node 6 was always chosen as MCH in phase 0 due to a slightly higher CENT metric. It is calculated from the ALM distances of all mesh paths and small ALM differences caused the preference of one node.
  In phase 1, the three remaining nodes were applied as PCHs since they shared the same NC and PCHNC metrics. Of these candidates, node 7 reproducibly remained as final CH in phase 2. Again, this was due to a slightly higher CENT metric, which serves as weighting factor in the WNPR metric. Our current concept allows CHs to emerge in close proximity to the MCH but not to each other. This is motivated by applications where the MCH serves administrative purposes even beyond cluster formation. Use cases include, e.g., the retrieval of status information (monitoring) of all nodes, which we outlined in a previous case study [17]. Here, on the one hand, proximity to the MCH is desirable for efficient data synchronization. On the other hand, the coverage of individual network regions by the CHs is targeted. After CH announcement in phase 4, node 1 and 2 always joined the cluster of the MCH, which is preferred over other neighboring CHs. This strategy also favors the monitoring scenario, in which the synchronization of status information of nodes already managed by the MCH can be omitted. In phases 5 and 6, the CHs performed their virtual channel selection and claimed channel 36 and 40 from the predefined pool, as expected. By reaching phase 7, the initial cluster formation was complete.
  The currently allowed emergence of CHs in neighborhood to the MCH causes clusters to be formed even in small topologies that do not require further subdivision (see 2 × 2 and 3 × 3 grids). However, as part of the evaluation, this behavior was intended to allow CHaChA to be studied in the laboratory setup. For practical use, CHs in close proximity to the MCH could be prevented or a minimum ALM distance could be mandated, depending on the overall node count or estimated network diameter. Similarly, a minimum network size could be defined above which cluster formation is performed at all.
**3 × 3-node grid:** In the topology with nine nodes, four different cluster constellations emerged (see Figure 15), with the central node 7 always reliably obtaining the MCH role. In the 3 × 3 grid, generally only nodes 2, 6, 8, and 12 were considered as additional CH candidates, as they had the highest number of neighboring nodes after node 7 (NC =5). In the most frequent constellation (Figure 15a, 7/10 measurements), nodes 6 and 8 each won the contest with nodes 2 and 12 due to dominant CENT metrics. In contrast, the remaining three constellations (Figure 15b–d, 1/10 measurements each) comprised only two clusters and differed in the position of the second CH. Due to variations in the ALM cost between nodes, from which the CENT value is derived as a weighting factor of the WNPR metric, in each of these measurements, a different pair of diagonally adjacent PCHs with dominant WNPR competed with each other, resulting in only a single CH being selected besides the MCH. As in the 2 × 2 setup, the remaining nodes preferentially joined the MCH cluster in all constellations. Balancing after reaching phase 7 would lead to a roaming of edge nodes into the still unoccupied clusters.
**4 × 4-node grid:** As in the 2 × 2 grid, no symmetric central node position existed in the 4 × 4 topology. Accordingly, the nodes of the inner square (nodes 7, 8, 12, and 13) were the expected candidates for the MCH role and another CH due to their dominant CENT metric and maximal neighbor count (NC =8). In the total of ten measurements, ALM variations led to nine different constellations (see Figure 16), which differed in the selected CH pair and / or the subsequent joining decisions of the CMs. While nodes adjacent to the MCH preferentially joined its cluster, nodes further away again made their decision based on the ALM costs to the CHs. As a result, balanced cluster sizes already emerged in constellations 4, 7, and 9 (Figure 16d,g,i). Even in constellation 6 (Figure 16f), the difference was only one node. Subsequent CM roaming would lead to a balancing of cluster sizes here as well.
**5 × 5-node grid:** As with parameterization P1 (see Section 6.2.3, Figure 13), the same most frequent cluster constellation was also formed with parameterization P2 in 9 of 10 measurements (see Figure 17a). Differences from the previous result existed only in the CH order during channel selection in phase 5, which depends on the ALM cost between the CHs and thus may be subject to slight metric variations. The constellation 2 shown in Figure 17b, which was formed in only one measurement, also resulted from an ALM-based decision. Here, node 11 did not consider CH 17 (red cluster) but CH 7 (purple cluster) as closest CH from its point of view in phase 4. Due to the more than doubled number of CHs in the 5 × 5 grid, compared to the previous topologies, as well as the majority of nodes being outside the neighborhood of the MCH, the initial phase sequence already formed clusters that differed in size only by a maximum of two nodes. Again, further balancing would lead to equal cluster sizes, as demonstrated for the 5 × 5 grid in Section 6.3.1.
**Comparison of clustering duration and communication overhead:** Following the functional validation, clustering duration and communication overhead were compared in all scenarios. Table 10 shows the average clustering duration as well as TCP/UDP data size and packet count for each topology. The results for the 5 × 5-node grid using parameterization P1 can be found in the last row of the table for comparison. Figure 18 and Figure 19 show the results for parameterization P2 including their standard deviation. For parameterization P2, the estimation described in Section 6.2.1 resulted in an expected clustering duration of 50 s. It should be emphasized that our estimation uses simplified assumptions for the time components $T_{NewCH}$, $T_{KO}$, and $T_{SelectChannel}$, which are in fact topology-dependent and subject to random processes. Of these, time $T_{NewCH}$ was omitted (as with parameterization P1) because the phase sequence was always started simultaneously on all nodes. In addition, with parameterization P2, the initial check for existing clusters in phase 0 was disabled. Contrarily, $T_{KO}$ (convergence time of broadcast-based MCH election in phase 0) as well as $T_{SelectChannel}$ (time required for CH communication during channel selection in phase 5) were responsible for the clustering duration increasing with network size and CH count. Furthermore, our estimation neglects any execution delays on the devices, and therefore also the impact of message processing times increasing with the number of nodes.
  In the 2 × 2- and 3 × 3-node grids, the influence and variability of the topology-dependent time components were still low, and the measured average clustering duration of 49.3 s and 50.5 s, respectively, was close to the estimated duration of 50 s (see Figure 19). As the number of nodes increased, the average duration slowly rose above the estimate. Clustering took 52.9 s in the 4 × 4 grid and 55.6 s in the 5 × 5 grid, respectively. At the same time, the standard deviation increased to approx. 1 s (4 × 4 grid) and 2 s (5 × 5 grid).
  In terms of communication overhead, the trends shown in Figure 18 were obtained for the TCP and UDP traffic, again showing a standard deviation increasing with node count. As expected, the TCP trend exhibited a nearly linear dependence on the number of nodes. The reason for this is that CHaChA performs reliable information exchange via TCP mainly between neighboring nodes. The only exceptions are JOIN messages sent by CFNs to CHs in phase 4 and CHAN_SEL messages exchanged between CHs in phase 5, where endpoints may be several hops apart. Consequently, TCP traffic share is less determined by the network diameter, but significantly by the total node count and the number of neighbors per node.
  Regarding UDP traffic, the expected dependence on grid size and CH count was confirmed. In the prototype implementation of CHaChA, UDP is used exclusively for broadcast messages that are propagated throughout the network. As the network diameter increases, the average path length between nodes increases as well, so more forwarding steps are caused by each broadcast. From phase 3 onwards, the periodic transmission of CH announcement messages is performed, which contributes significantly to the UDP traffic share. With increasing network diameter, there are potentially more PCH candidates, potentially resulting in more final CHs. For example, between the 4 × 4 and 5 × 5 grids, there is a rise from two to five CHs, causing a corresponding increase in UDP traffic.
  For both UDP and TCP, the data size trend corresponds to that of the packet count (see right diagram in Figure 18), including any multi-hop forwarding and TCP control messages. This also accounts for the larger increase in TCP traffic compared to UDP, as TCP generates additional handshake and ACK messages. In the 5 × 5-node grid, TCP data size and packet count amounted to 3–4 times the UDP results. In the topologies studied, the increase in individual packet lengths had only little effect on the total data size. Besides the CHAN_SEL messages in phase 5, carrying the list of CHs involved, the periodic CH broadcast announcements are the only messages with their size depending on the network topology, since they contain the list of CMs in the respective cluster.
  The average total TCP/UDP data size of 2526 kB, generated for parameterization P2 in the 5 × 5 grid over an average clustering duration of 55.6 s, can again be simplified as overhead traffic with an effective data rate of only 360 kbit/s. Basically, a trade-off between clustering duration and communication cost has to be found through parameterization. For a low long-term overhead beyond the phase sequence, the choice of a suitable transmission period for the CH broadcast messages is particularly necessary. At the same time, this should preserve the responsiveness of the dynamic cluster adaptation mechanisms, where decisions are partly based on the information provided by the CH announcements. A general reduction of message sizes could be achieved in the future by a more compact encoding or by means of compression methods. Furthermore, depending on the desired degree of reliability, the use of UDP is also conceivable for unicast messages between neighboring nodes.

### 6.3. Assessment of Online Cluster Adaptation

After detailed analysis of the CHaChA phase sequence for initial clustering, the concepts for dynamic cluster adaptation in long-term operation, described in Section 4.4, were also functionally tested in example scenarios. For this, we kept parameterization P2 of the prototype implementation (see Table 9). By specifying an initial cluster constellation, all nodes immediately jumped to phase 7 at program start, where the cluster adaptation mechanisms took effect.

In addition to the offline visualization of cluster constellations from the status files of the nodes, it was helpful for the subsequent experiments to obtain and display current cluster information at runtime. A Java application developed for this purpose was run on a separate lab PC and generated an image of the current cluster status based on messages received over the control network. To achieve this, all CHs periodically sent their cluster information (WLAN channel and list of CMs) not only as broadcast in the mesh network, but simultaneously as unicast via Ethernet to the PC. Hence, the freshness of cluster information on the nodes and the PC was given by the broadcast interval of the CH messages (CH_PERIOD). In the experiments, all devices were started simultaneously. As for the offline visualization, the grid positions of the nodes were again predefined. Based on received information about their role and cluster membership, nodes were highlighted accordingly. To diagnose the cluster adaptation mechanisms, videos of the desktop application were recorded during experiment execution. Shown below are snapshots of the times when cluster changes became visible in the application (time resolution of 1 s).

#### 6.3.1. Cluster Balancing through Roaming

In a first set of experiments, the subsequent cluster balancing by autonomous roaming of CMs was investigated. As described in Section 4.4.3 and Section 4.4.4, CMs make their roaming decisions based on the current cluster information received via periodic CH broadcast messages. Thereby, only CMs at the cluster edge, which are not connecting their CH to other members of their cluster, are eligible for roaming. To determine this, from phase 7 onwards, all CMs exchange NH2CH unicast messages periodically with their neighbors. These contain information about which neighbor serves as next hop on the mesh path to the CH, so that each CM can check its suitability for a cluster change. In all experiments, the NH2CH broadcast interval was chosen to be identical to the broadcast period of the CH messages (2 s for parameterization P2). Since our testbed devices were equipped with only one WLAN adapter, only NH2CH information of the primary interface was exchanged. In this setup, it is theoretically possible that the path to the own CH could also pass through nodes of a neighboring cluster. However, in the example studied, all CMs were in direct proximity to their CH, so that paths were always created without intermediate nodes. Consequently, all CMs at the edge to other clusters were able to perform roaming operations. According to the balancing concept (see Section 4.4.4), CMs at the cluster edge only initiate a roaming request if the size of their cluster is at least 2 nodes above that of a neighboring cluster. In the course of handshaking with old and new CH, simultaneous migrations (within a CH_PERIOD) of multiple CMs from the same source to the same destination cluster are prevented to avoid avalanche effects.

**Figure 20:**
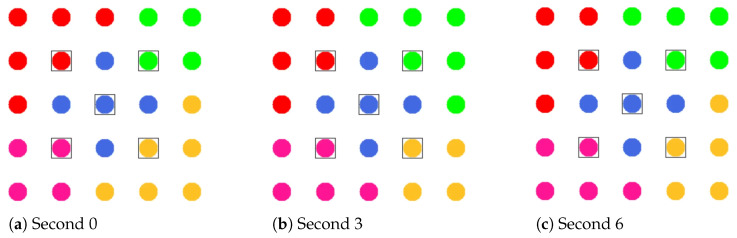
Cluster balancing in the 5 × 5-node grid ((**a**): constellation after initial clustering, (**b**): constellation after one roaming cycle, (**c**): balanced constellation after two roaming cycles). Reprinted from ref. [18].

Figure 20 shows the course of an experiment in the 5 × 5-node grid, in which the cluster constellation formed most frequently with parameterizations P1 and P2 was given as initial configuration. The five CHs are each marked with a black square, with the MCH in the center of the grid not highlighted individually. As in the offline visualization, all members of a cluster are shown in the same color. Immediately after initialization of the constellation in “second 0”, the red and yellow clusters were each two nodes larger than the purple and green clusters. Only the central blue cluster already corresponded to the targeted average size of five nodes. In second 3 of the experiment, it became apparent that one CM of the red cluster had switched to the green cluster. Independently, two CMs of the yellow cluster had simultaneously switched to the green and purple clusters, respectively. This was allowed since they were different target clusters, but now resulted in an excess CM in the green cluster. Subsequently, the CM that had previously changed to the green cluster roamed back to the yellow cluster, completing the balancing in second 6.

In this example, the initial constellation was already close to the balancing target and only a few roaming steps were needed to align the cluster sizes. Convergence was reached after only two roaming cycles, with the timing of CM decisions coinciding with the broadcast interval of CH messages. In larger topologies that exhibit more imbalance after initial cluster formation, significantly more roaming steps may be required for balancing. Allowing parallel roaming operations, such as that of the CMs of the yellow cluster between second 0 and 3, can thereby influence the convergence time either positively or negatively, thus providing scope for further optimization.

#### 6.3.2. Addition and Relocation of Nodes

Further experiments were dedicated to a network maintenance scenario in which a combination of the mechanisms for dynamic cluster adaptation was triggered. We investigated the addition (subsequent installation) of a node into an existing cluster constellation. First, it required automatic cluster joining of the node according to the concept in Section 4.4.1. This was followed by a (virtual) relocation of the node until its connection to the original CH was lost. Isolation detection (concept in Section 4.4.2) on the node then resulted in joining a new cluster, which in turn caused cluster balancing by roaming of other nodes (concept in Section 4.4.4). In the context of urban mesh backbones, addition and relocation of nodes are expected measures, e.g., to increase coverage or resilience of the network.

**Figure 21:**
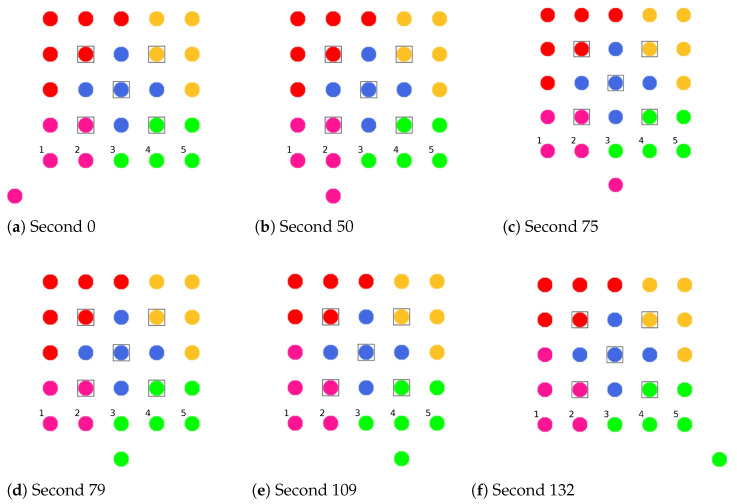
Addition and virtual relocation of a node ((**a**): added node joined purple cluster, (**b**,**c**): virtual relocation, (**d**): node joined green cluster, (**e**,**f**): virtual relocation and balancing). Reprinted from ref. [18].

During our experiment, the new node was permanently placed next to the test environment. Unlike the other devices, its antennas were not equipped with attenuators, allowing a direct connection to any other node. However, we blocked link establishment to all but one of the nodes 1–5 in Figure 21 via Linux WLAN interface configuration. Starting at node 1, the virtual relocation of the additional node along the bottom edge of the network was done by blocking the previous link and allowing the next one, respectively. Analogous to the broadcast messages of the CHs, the additional node sent its NH2CH messages to the control PC at 2 s intervals both in the mesh network and via Ethernet. Based on the information about which of the nodes 1–5 currently served as next hop to the CH, the position of the “relocated” node was updated in the visualization software.

Figure 21 shows the results of an experiment execution. The most frequently formed cluster constellation of the 5 × 5-node grid topology again served as initial configuration. Otherwise keeping parameterization P2, the check for existing clusters in phase 0 was now activated on the additional node. After initialization of the grid, the delayed program start of the additional node was preceded by an automatic balancing process between the green and yellow clusters. However, the balancing between the red and violet clusters, which was also pending, had not yet taken place. When starting, the additional node already had a connection to the network via node 1. After evaluating the incoming CH announcements in phase 0, it chose to join the purple cluster as its CH had the smallest ALM distance. This created an initially balanced cluster constellation.

Second 0 in Figure 21 shows the time when the additional node had successfully joined the network, and its position and cluster membership were represented in the visualization according to the received CH and NH2CH messages. From this point on, the control PC performed the sequential “relocation” of the node. Switching the connection to the next of the nodes 1–5 in each case was done script-based in intervals of 30 s.

In second 50, i.e., 20 s after link handover to node 2, the position change appeared in the visualization. This time delay is partly due to the parameterization of the prototype and the 802.11s link layer, and partly due to the delivery probability of 802.11s management and control frames. According to the CHaChA concept, CHs use the proactive mode of the 802.11s routing protocol HWMP, so that all nodes always maintain path information to them. The sending interval of the proactive PREQ frames for the path update is configurable via the 802.11s parameter HWMProotInterval. For the purposes of this work, its default value of 5 s was kept (see [1,47] for an overview of 802.11s parameters). Although nodes usually detect link breaks after one beacon frame period (default value 1 s), the update of the path entry to the CH, whose next-hop information may have become obsolete, does not occur until the next PREQ frame is received. Since PREQs are always sent as unacknowledged broadcasts, they have a lower delivery probability compared to unicast frames. The loss of a PREQ frame thus delays the path update by another 5 s.

After link switching on the additional node, it must detect the path update not only at the link layer but also in the CHaChA application before it can send the current NH2CH information to the control PC. In our prototype, the 802.11s information of the Linux kernel is sampled every 2 s. Depending on the offset of the PREQ interval (5 s) and sampling cycle, a path change is thus detected after 6–8 s in the node’s application and visualized after another 2 s according to the transmission interval of the NH2CH messages. In addition, there are delays due to undelivered PREQ messages. In the experiment discussed here, link changes appeared in the visualization after 12–20 s.

Second 75 in Figure 21 shows the detected link switch from node 2 to node 3, which had been triggered in second 60. By crossing the cluster boundary, the isolation detection of the new node detected the link loss to the CH of the purple cluster, initiating a change to the green cluster. Since only one WLAN adapter (primary interface) was used in all experiments and all nodes were connected on the base channel, the path to the purple CH could still be established through the CM of another cluster. Thus, isolation detection was not possible based on information from the secondary interface that would signal a lost path to the CH on the cluster channel. Deviating from the concept in Section 4.4.2, detection was instead performed by checking whether the path to the current CH passes through a CM of the same cluster. The members of all clusters are announced in the CH broadcasts in the network and were thus also known to the new node. Successful joining of the green cluster became apparent in second 79, after fresh CH messages of all clusters had been sent to the control PC. The now reduced number of nodes in the purple cluster subsequently caused the roaming of an edge node from the red cluster, resulting in re-balancing of the constellation. As shown in Figure 21, the link switches to nodes 4 and 5 appeared in the visualization after 19 and 12 s, respectively.

In summary, the concepts for subsequent network joining as well as isolation detection and cluster balancing were successfully tested in combination. While the focus of these experiments was on functional validation, the choice of shorter update intervals (CHaChA and 802.11s parameters) can increase responsiveness to network changes. Depending on the use case, a compromise must be found between latency and messaging overhead.

## 7. Discussion and Conclusions

In this work, we present the design of CHaChA, a decentralized clustering and channel selection solution for WLAN mesh networks that specifically takes into account the mesh features introduced in the standard amendment IEEE 802.11s. It uses only standard mechanisms of the 802.11s specification and does not require any modification at the WLAN link layer. The default 802.11s information, retrievable via existing system interfaces on every node, are used to derive the clustering metrics for our approach.

Our main contribution is a phase-based algorithm for cluster formation in WLAN mesh backbones, whose nodes nowadays usually have two or more physical WLAN interfaces. Of these, one remains on a dedicated base channel, which always ensures best possible connectivity and serves CHaChA coordination. A secondary interface allows cluster-internal communication on orthogonal channels, which relieves the base channel and reduces interference. Moreover, we present mechanisms for automatic cluster adaptation to topology changes. These include subsequent or repeated cluster joining, mutual isolation and failure detection of nodes, as well as node roaming for cluster balancing.

As a cross-layer approach, CHaChA integrates inherently available link and path information of the 802.11s link layer into the application layer to derive topology information and use it for cluster formation and channel selection. In turn, network structure optimization is performed at the application layer by configuring secondary interfaces. The selective instrumentation of standard mechanisms, such as the 802.11s routing protocol HWMP, plays an important role. The use of different variants of the proactive HWMP mode enables, on the one hand, a low-cost node discovery compared to mechanisms at higher protocol layers and, on the other hand, the mutual failure detection between nodes.

By investigating our prototype implementation in a real-world testbed using grid topologies with up to 25 nodes, the practicality of the approach was demonstrated. With an occurrence rate of over 90 % for the most frequent cluster constellation in the 5 × 5-node grid, the experiments prove the reproducibility of the results. A conservatively chosen initial parameterization of our algorithm was experimentally adjusted to the test environment, resulting in a clustering duration below one minute in the 5 × 5-node grid and a sent data volume of approx. 2.5 MB. The realization of the algorithm as a phase sequence facilitates its practical analysis and allows time estimates that correspond well with the measured clustering duration.

In different grid sizes, we investigated the dependence of the amount of data sent on the number of nodes. As expected, an almost linear relationship was found for TCP, which is mainly used for communication between neighboring nodes in our concept. In the case of UDP, a stronger nonlinear topology dependency was shown, since it is used for network-wide broadcast messages. Beyond the initial cluster formation, however, the long-term communication overhead is dominated in particular by periodic CH announcements, whose transmission interval can be chosen depending on the use case. Finally, we practically validated our mechanisms for subsequent network joining, fault detection, node roaming, and cluster balancing, which are essential for long-term network operation.

Future studies could focus on automatically deriving and optimizing 802.11s and CHaChA protocol parameters for different network sizes, device classes, and use case requirements. In this context, we intend to evaluate CHaChA in larger and more diverse network scenarios. Prospectively, employing a more powerful device platform in our testbed will also allow the use of higher 802.11 data rates and enable further fine-tuning of the timing parameters of our algorithm. As an additional goal, we plan to conduct a quantitative comparison with the related works, as discussed in Section 3. This, however, requires practical (re-)implementation of the approaches, which have so far only been evaluated using simulations.

## Figures and Tables

**Table 1:** Comparison of CHaChA with related works (*: preliminary or modified 802.11s without ALM). Adapted with permission from ref. [17]. © 2018 IEEE.

Works	Consideration of 802.11s	Distributed Approach	Real Testbed
CCAS [26], ISC [27], MCI [28], MCCA [29]	—	—	—
CCA [30], CoMTaC [31], DCITCA [32], Max-Min-D [33]	—	✓	—
CGCA [9]	*	—	—
Kapse et al. [10], JRCAP [11]	*	✓	—
CHaChA [17]	✓	✓	✓

**Table 2:** Node roles in CHaChA. Reprinted from ref. [18].

Role	Abbreviation	Description
Cluster-Free Node	CFN	starting role, node without cluster
Cluster Member	CM	node belonging to a cluster
Cluster Head	CH	node organizing a cluster
Proposed Cluster Head	PCH	temporary CH candidate in phase sequence
Master Cluster Head	MCH	phase coordinator and first selected CH

**Table 3:** Metrics in CHaChA ($c_{a}$: airtime cost, *O*: 802.11 protocol overhead, *B*: frame bit length, *r*: 802.11 data rate, *e*: frame error probability). Reprinted from ref. [18].

Metric	Abbreviation	Calculation
Airtime Link Metric	ALM	per hop: $c_{a}=\left(O+\frac{B}{r}\right)\cdot \frac{1}{1-e}$ per path: $ALM=\sum_{i=1}^{\#Hops}c_{a_{i}}$
Centrality	CENT	$CENT={\left(\sum_{i=1}^{\#Paths}ALM_{i}\right)}^{-1}$
Network Size	N	N=1+#(Paths)
Neighbor Count	NC	NC=#(Links)
PCH Neighbor Count	PCHNC	PCHNC=#(LinkstoPCHs)
NC-to-PCHNC Ratio	NPR	$NPR=\frac{NC}{(1+PCHNC)\cdot N}$
Weighted NPR	WNPR	$WNPR=NPR\;\cdot \frac{CENT}{CENT_{max}}$
Integer value of MAC address	—	Example: 00:00:00:00:fa:00 →64,000

**Table 4:** Unicast (UC) and broadcast (BC) messages in CHaChA. Reprinted from ref. [18].

Type (Opcode)	UC/BC	Description
CENT	BC	network-wide dissemination of CENT metric, sent by CFNs in phase 0
NC	UC	announcement of NC metric to neighbors, sent by CFNs in phase 0
PCH	UC	announcement of PCH role to neighbors, sent by PCHs in phase 1
WNPR	UC	exchange of WNPR metric between neighboring PCHs in phase 2
CH	BC	periodic announcement of cluster info (mesh ID, channel, list of CM MAC addresses), sent by CHs from phase 3 onwards
JOIN	UC	control message for cluster joining, sent by CFNs (CMs)
LEAVE	UC	control message for cluster leaving, sent by CMs
CHAN_SEL	UC	control message for channel selection, exchanged between CHs in phase 5; carries pairs of CH MAC address and channel number
PHASE_X	BC	MCH announcement of transition to phase X (defined for phases 1–6)
NH2CH	UC	exchange of Next-Hop-to-CH(NH2CH) info between neighboring CMs in phase 7; used for roaming and cluster balancing (see Section 4.4.4)
JOIN_REQ/RESP LEAVE_REQ/RESP	UC	handshake messages between CMs and CHs to coordinate roaming operations in phase 7 (see Section 4.4.3)

**Table 5:** Timing parameters and thresholds in CHaChA (# indicates integer value without unit). Reprinted from ref. [18].

Parameter	Unit	Description
CENT_PERIOD	ms	sending interval of CENT messages in phase 0
CENT_THRESH	#	max. no. of CENT messages sent without receiving any; exceeded by winner of K.O. race for MCH role in phase 0
NC_PERIOD	ms	sending interval of NC messages in phase 0
CH_PERIOD	ms	sending interval of CH messages from phase 3 onwards
CH_THRESH	#	max. no. of CH_PERIODs to wait for incoming CH messages in phase 0 (detection of existing clusters)
PHASE_X_DELAY	ms	waiting time in phase X-1 before MCH announces phase X (defined for phases 2–5)
PHASE_X_PERIOD	ms	sending interval of PHASE_X messages (defined for phases 1–6)
PHASE_X_TRIES	#	no. of repetitions of PHASE_X messages (defined for phases 1–6)
PHASE_X_TIMEOUT	ms	timeout of transition to next phase (defined for phases 1–6); exceeding leads to switching back in phase 0
NH2CH_PERIOD	ms	sending interval of NH2CH (Next-Hop-to-CH) messages in phase 7
CONN_TIMEOUT	ms	timeout of recovery from error state (connection loss) in phase 7; exceeding leads to switching back in phase 0

**Table 6:** Structure of CHaChA unicast (UC) and broadcast (BC) messages. Reprinted from ref. [18].

Type (Opcode)	UC/BC	Payload of an Example Message
CENT	BC	“CENT|0.0021”
NC	UC	“NC|8”
PCH	UC	“PCH”
WNPR	UC	“WNPR|0.1875”
CH	BC	“CH|mesh_id|36|00:00:00:00:00:AA|...|00:00:00:00:00:AF”
JOIN	UC	“JOIN”
LEAVE	UC	“LEAVE”
CHAN_SEL	UC	“CHAN_SEL|00:00:00:00:00:A1|36|...|00:00:00:00:00:A5|153”
PHASE_X	BC	“PHASE_1”
NH2CH	UC	“NH2CH|00:00:00:00:00:AA”
JOIN_REQ/RESP LEAVE_REQ/RESP	UC	“JOIN_REQ” → “JOIN_RESP|1” (ACK) or “JOIN_RESP|0” (NACK) “LEAVE_REQ” → “LEAVE_RESP|1” (ACK) or “LEAVE_RESP|0” (NACK)

**Table 7:** Testbed configuration.

Parameter	Value
Device	Intel Galileo Board (Gen. 1)
CPU	Quark X1000 (single-core 400 MHz)
RAM	256 MB DDR3
OS	Debian 8 (Linux Kernel v4.9)
802.11 NIC	Compex WLE200NX 802.11a/b/g/n (mPCIe)
NIC Chipset	Atheros AR9280 (ath9k driver)
Antennas	2 × 5 dBi dual-band omni-directional
Attenuators	2 × Mini-Circuits VAT-30+ (30 dB)
Default Channel	149 (5745 MHz, HT20, Long GI)
TX Rate	HT MCS 3 (16-QAM 1/2, 26 Mbps)
TX Power	14 dBm (25 mW)

**Table 8:** CHaChA parameterization P1 (# indicates integer value without unit). Reprinted from ref. [18].

Parameter	Unit	Value
CENT_PERIOD	ms	500
CENT_THRESH	#	20
NC_PERIOD	ms	5000
CH_PERIOD	ms	5000
CH_THRESH	#	2
PHASE_DELAY	ms	10,000
PHASE_PERIOD	ms	500
PHASE_TRIES	#	20

**Table 9:** CHaChA parameterization P2 (# indicates integer value without unit; changes to variant P1 are bold). Reprinted from ref. [18].

Parameter	Unit	Value P2	Value P1
CENT_PERIOD	ms	500	500
CENT_THRESH	#	**10**	20
NC_PERIOD	ms	**2000**	5000
CH_PERIOD	ms	**2000**	5000
CH_THRESH	#	**0**	2
PHASE_DELAY	ms	**2000**	10,000
PHASE_PERIOD	ms	500	500
PHASE_TRIES	#	**10**	20

**Table 10:** Comparison of clustering duration and communication overhead (# indicates “number of”). Reprinted from ref. [18].

Topology	Estimated Duration [s]	Measured Duration [s]	TCP Data Size [kB]	# TCP Packets	UDP Data Size [kB]	# UDP Packets
2 × 2-node grid (P2)	50	49.3	131.3	1131	44.2	374
3 × 3-node grid (P2)	50	50.5	472.8	4069	105.2	835
4 × 4-node grid (P2)	50	52.9	1156.6	9952	191.5	1498
5 × 5-node grid (P2)	50	55.6	1929.2	16,695	597.2	4133
5 × 5-node grid (P1)	130	135.5	1113.7	9699	627.8	5248

## Abbreviations

The following abbreviations are used in this manuscript:

ALM	Airtime Link Metric
CENT	Centrality
CFN	Cluster-Free Node
CHaChA	Clustering Heuristic and Channel Assignment
CH	Cluster Head
CM	Cluster Member
HWMP	Hybrid Wireless Mesh Protocol
MAC	Media Access Control
MCH	Master Cluster Head
MCS	Modulation and Coding Scheme
NH2CH	Next-Hop-to-Cluster-Head
NC	Neighbor Count
NPR	NC-to-PCHNC-Ratio
PCH	Proposed Cluster Head
PCHNC	Proposed Cluster Head Neighbor Count
PREP	Path Reply
PREQ	Path Request
RANN	Root Announcement
TCP	Transmission Control Protocol
UDP	User Datagram Protocol
WLAN	Wireless Local Area Network
WMN	Wireless Mesh Network
WNPR	Weighted NC-to-PCHNC-Ratio
